# MicroRNA-3148 acts as molecular switch promoting malignant transformation and adipocytic differentiation of immortalized human bone marrow stromal cells via direct targeting of the SMAD2/TGFβ pathway

**DOI:** 10.1038/s41420-020-00312-z

**Published:** 2020-09-01

**Authors:** Radhakrishnan Vishnubalaji, Ramesh Elango, Muthurangan Manikandan, Abdul-Aziz Siyal, Dalia Ali, Ammar Al-Rikabi, Dana Hamam, Rimi Hamam, Hicham Benabdelkamel, Afshan Masood, Ibrahim O. Alanazi, Assim A. Alfadda, Musaad Alfayez, Abdullah Aldahmash, Moustapha Kassem, Nehad M. Alajez

**Affiliations:** 1 Cancer Research Center, Qatar Biomedical Research Institute (QBRI), Hamad Bin Khalifa University (HBKU), Qatar Foundation (QF), Doha, Qatar; 2 Stem Cell Unit, Department of Anatomy, College of Medicine, King Saud University, Riyadh, Saudi Arabia; 3 Molecular Endocrinology Unit (KMEB), Department of Endocrinology, University Hospital of Odense and University of Southern Denmark, Odense, Denmark; 4 Department of Pathology, King Saud University Medical City, Riyadh, Saudi Arabia; 5 McGill University Health Centre and RI-MUHC, Montreal, QC Canada; 6 Departement of Medicine, University of Montreal, Montreal, QC Canada; 7 Proteomics Resource Unit, Obesity Research Center, College of Medicine, King Saud University, Riyadh, 11461 Saudi Arabia; 8 The National Center for Biotechnology (NCBT), Life Science and Environment Research Institute, King Abdulaziz City for Science and Technology (KACST), Riyadh, Saudi Arabia; 9 Department of Cellular and Molecular Medicine, Danish Stem Cell Center (DanStem), University of Copenhagen, 2200 Copenhagen, Denmark

**Keywords:** Oncogenes, miRNAs

## Abstract

MicroRNAs (miRs/miRNAs) play a key role in posttranscriptional regulation of gene expression and are implicated in a number of physiological and pathological conditions, including cellular malignant transformation. In the current study, we investigated the role of miR-3148 in regulating human stromal (mesenchymal) stem cell (hMSC) differentiation and transformation. Stable expression of miR-3148 in telomerized hMSC (hMSC-miR-3148) led to significant increase in in vitro adipocytic differentiation and suppression of osteoblastic differentiation. Concordantly, global gene expression profiling revealed significant enrichment in cholesterol biosynthesis pathway, and pathways related to enhanced cell movement and survival, whereas processes related to bone and connective tissue developments, cell death, apoptosis, and necrosis were downregulated. Global proteomic analysis using 2D-DIGE followed by mass spectrometry (MS) revealed significant changes in protein expression in hMSC-miR-3148 and enrichment in protein networks associated with carcinogenesis. Functional studies revealed that hMSC-miR-3148 exhibited enhanced in vitro cell proliferation, colony formation, migration, invasion, sphere formation, doxorubicin resistance, and increased active number of cells in S and G2/M cell cycle phases and formed sarcoma-like tumors with adipocyte infiltration when implanted into immunocompromised mice. SMAD2 was identified as bone fide gene target for miR-3148 using qRT-PCR, Western blotting, and UTR-based reporter assay. In agreement with our data, SMAD2 expression was downregulated in 47% of patients with soft tissue sarcoma. Bioinformatics analysis revealed that elevated miR-3148 expression correlates with poor prognosis in several human cancer types, including sarcoma. Our study identified miR-3148 as factor regulating hMSC differentiation and is involved in promoting malignant transformation of telomerized hMSC.

## Introduction

MicroRNAs (miR/miRNA) are regulatory noncoding double-stranded RNAs that exert posttranscriptional regulation of gene expression under normal and pathological conditions^1^. Global transcriptomic analysis of miRNAs in cancer tissue compared with normal tissue revealed differential expression of a number of miRNAs which are either downregulated in cancer and thus exert a tumor suppressor role through direct targeting of oncogenes or upregulated miRNAs, which act as oncomiRs through inhibition of tumor suppressor genes^2^. Deregulated miRNA expression is associated with large number of human cancers, including breast, colon, gastric, lung, and sarcomas^3–8^. MiRNAs have also been implicated in regulating epithelial-to-mesenchymal transition, a process leading to endowment of cancer cells with a mesenchymal phenotype^9^.

MiRNAs have also been implicated in regulating stem cell differentiation^10^. For instance, we have previously shown the miR-320 family to promote adipocytic and to inhibit osteoblastic differentiation of human bone marrow stromal (mesenchymal) stem cells (hBMSCs) through repression of RUNX2 expression^11^. Interestingly, we also identified the miR-320 family to suppress colorectal cancer, through direct targeting of FOXM1, FOXQ1, and the stem cell marker, SOX4^12^, suggesting that miRNAs that play a role in regulating stem cell functions, may be associated with cancer development^13^.

In the current study, we investigated the biological effects of miR-3148, identified from our previous microRNA profiling during hMSC differentiation, on hMSC cell differentiation into osteoblast and adipocytes. Functional and molecular investigations revealed miR-3148 to promote adipogenesis and to suppress osteogenesis through direct targeting of SMAD2/TGFβ pathway. In addition, hMSC-miR-3148 exhibited enhanced in vitro cell proliferation, colony formation, cell migration, cell invasion and sphere formation, increased active S and G2/M cell cycle phases, and formed sarcoma-like tumors when implanted into immunocompromised mice, suggesting a role for miR-3148 as regulator of stem cell differentiation and transformation.

## Results

### MiR-3148 overexpression enhances adipocytic and suppresses osteoblastic differentiation

**Fig. 1:**
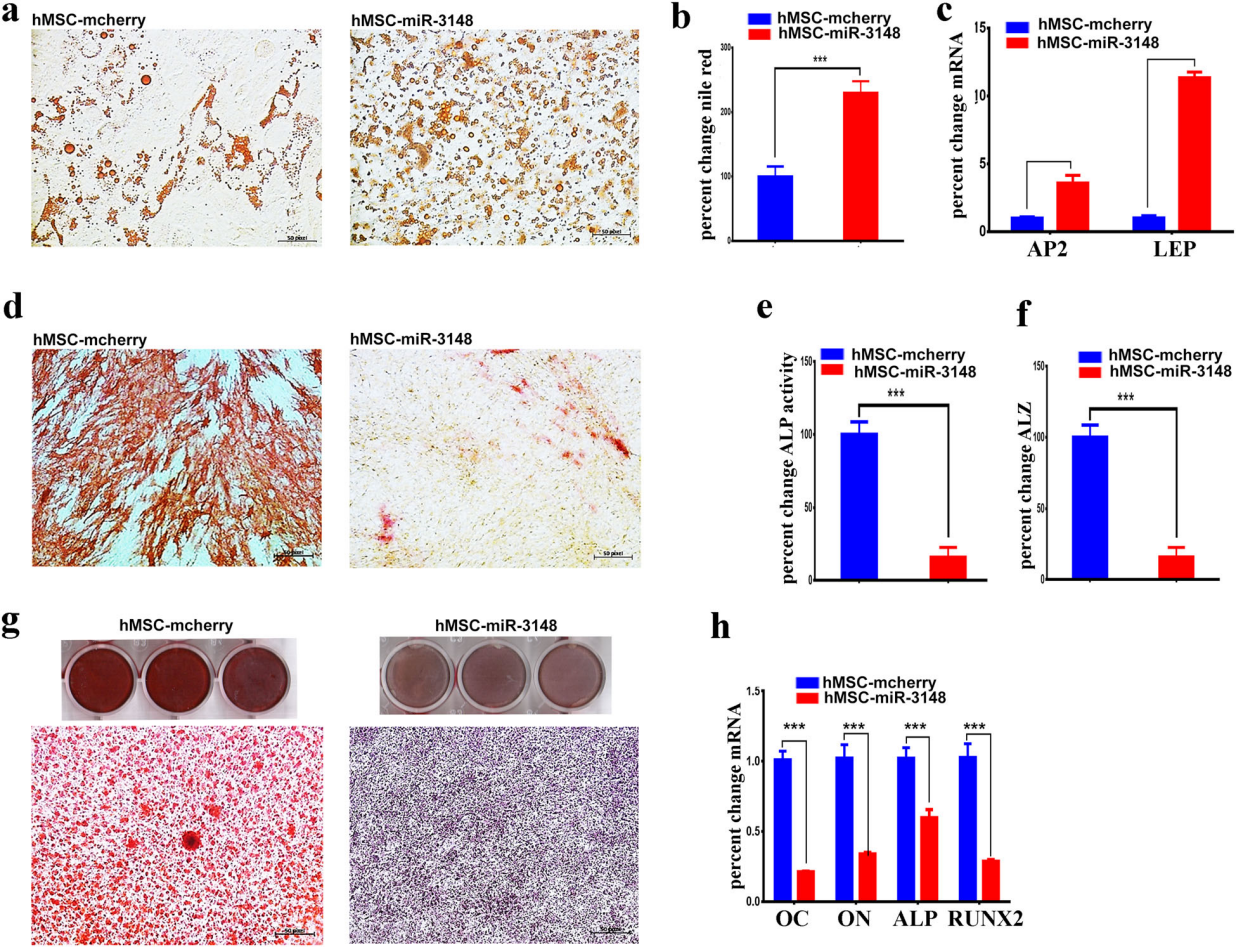
hMSC-miR-3148 cells exhibited enhanced adipocytic and reduced osteoblastic differentiation of hMSC. **a** Representative Oil Red-O staining on day 7 showing increased adipogenesis in hMSC-miR-3148 cells. Quantification of nile red staining (**b**) and adipocyte-specific gene marker (AP2 and LEP) expression (**c**) in hMSC-miR-3148 vs hMSC-mcherry cells. **d** Representative ALP staining of osteoblast cells on day 10 in hMSC-miR-3148 vs hMSC-mcherry cells. Quantification of ALP activity (**e**) and alizarin red (ALZ, **f**) in hMSC-miR-3148 vs hMSC-mcherry cells. **g** Representative images of alizarin red staining of mineralized matrix in hMSC-miR-3148 vs hMSC-mcherry cells performed on day 21. **h** Quantification of osteoblast specific gene markers (OC, ON, ALP, and RUNX2) in hMSC-miR-3148 compared to hMSC-mcherry using qRT-PCR. Data are presented as mean ± S.E.M. from three independent experiments, ***p < 0.0005.

Our previous global miRNA profiling identified miR-3148 as significantly downregulated miRNA during osteoblastic differentiation of human stromal (mesenchymal) stem cells (Supplementary Fig. 1). To unravel potential role for miR-3148 during stem cell differentiation, hMSC cells were transduced with a lentiviral vector encoding for miR-3148 (hMSC-miR-3148) which exhibited significant upregulation of miR-3148 expression compared to hMSC-control cells (Supplementary Fig. 2). Subsequently, hMSC-miR-3148 and hMSC-control cells were subjected to adipocytic and osteoblastic differentiation conditions. An enhanced adipocytic differentiation was observed in hMSC-miR-3148 as evidenced by oil red staining (Fig. 1a), Nile red quantification (Fig. 1b), and quantitative reverse transcriptase-polymerase chain reaction (qRT-PCR) for adipocyte protein 2 (AP2) and leptin (LEP, Fig. 1c). On the other hand, hMSC-miR-3148 exhibited diminished ALP activity (Fig. 1d, e), decreased in vitro mineralization (Fig. 1f, g) and decreased expression of osteoblastic gene markers: osteocalcin (OC), osteonectin (ON), alkaline phosphatase (ALP), and Runt-related transcription factor 2 (RUNX2, Fig. 1h).

### miR-3148 regulates multiple genetic pathways in hMSC

**Fig. 2:**
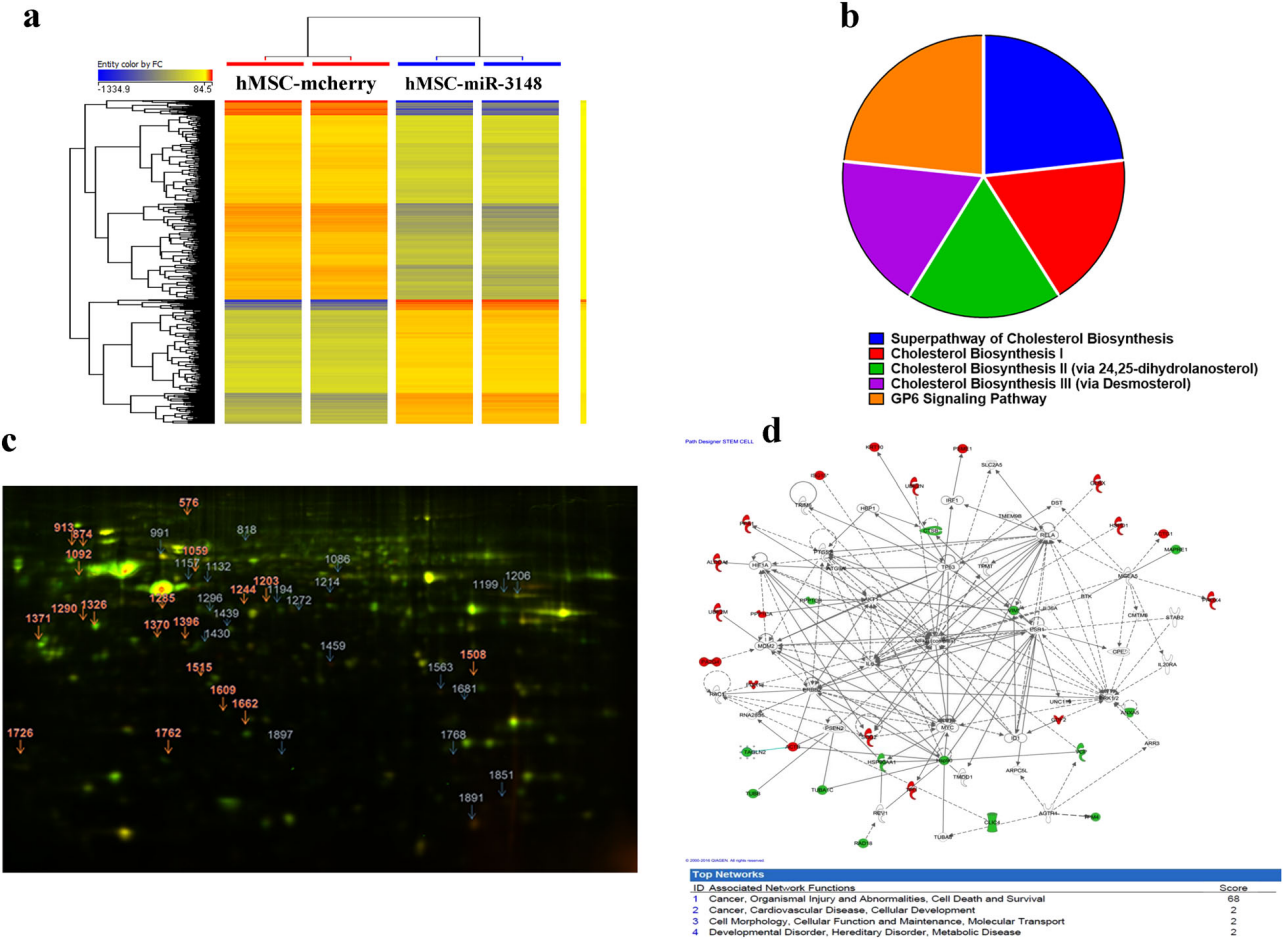
The hMSC-miR-3148 cell line exhibited multiple altered genetic pathways. **a** Hierarchical clustering of hMSC-miR-3148 compared to hMSC-mcherry cells based on differentially expressed mRNA levels as determined by microarray analysis. Each column represents one replica and each row represents an mRNA transcript. The expression level of each gene in a single sample is depicted according to the color scale. **b** Pie chart illustrating the distribution of the top-5 canonical pathways based on Ingenuity Pathway Analysis (IPA) on the upregulated genes in the hMSC-miR-3148 compared to hMSC-mcherry cells. The size of the chart segment corresponds to activation *Z* score. **c** Representative 2D-DIGE gel image. The arrows indicate differentially regulated protein spots between hMSC-miR-3148 and hMSC-mcherry cells as determined by image analysis and identified by MALDI-MS. The gray arrows indicate the upregulated and the red arrows indicate the downregulated proteins in hMSC-3148 cells. **d** Schematic representation of the “Cancer, Organismal injury and abnormalities, cell death and survival” functional interaction network with the highest score of 68 showing NFkB and IL6 as central nodes. Nodes in green and red correspond to down and upregulated proteins, respectively. Noncolored nodes are proposed by IPA and suggest potential targets functionally coordinated with the differential proteins. Solid lines indicate direct molecular interactions and dashed lines represent indirect relationships.

To gain more insight into the molecular mechanisms by which miR-3148 exert its effects during hMSC differentiation, we performed global transcriptome analysis of hMSC-miR-3148 compared to hMSC-control cells and identified 1476 upregulated and 2370 downregulated genes (2.0-fold change, *p* < 0.05, Supplementary Table 1) in the hMSC-miR-3148. Hierarchical clustering showed clear separate clustering of the hMSC-miR-3148 and hMSC-control cells (Fig. 2a). The list of upregulated genes were subsequently subjected to Ingenuity® Pathway Analysis (IPA). Data presented in Fig. 2b depict the top five enriched canonical pathways enriched in hMSC-miR-3148 cells and included cholesterol biosynthesis. Analysis for functional categories revealed significant enrichment in several categories, including cellular movement, cellular invasion, and cellular survival, while categories related to cell death, apoptosis, and necrosis were significantly underrepresented (Supplementary Fig. 3). The cellular movement and cellular death and survival functional categories are illustrated in Supplementary Fig. 3 as well. IPA analysis on the downregulated genes in hMSC-miR-3148 cells reveled significant downregulation in tissue development functional categories (Supplementary Fig. 3). Upstream regulator analysis of the differentially expressed genes revealed significant enrichment in a number of networks driven by Mitogen-Activated Protein Kinase 1 (MAPK1), Interleukin 1 Receptor Antagonist, Triggering Receptor Expressed On Myeloid Cells 1, Activating Transcription Factor 4, Bruton Tyrosine Kinase, TGF-Beta Activated Kinase 1 (MAP3K7) Binding Protein 1 (TAB1), Amphiregulin (AREG), CCR4-NOT Transcription Complex Subunit 7 (CNOT7), Vascular Endothelial Growth Factor A, and TAL BHLH Transcription Factor 1, Erythroid Differentiation Factor (TAL1) (Supplementary Fig. 3).

In addition, we performed global proteomic analysis using 2D-DIGE followed by protein identification by mass spectrometry (MS). Proteomic analysis identified 2000 spots consistently mapped to all gels (Fig. 2c). Sixty protein spots exhibited statistically significant change in abundance in hMSC-miR-3148 compared to hMSC-control cells (ANOVA-test *p* < 0.05; fold change > 1.5). These spots were picked and further analyzed by MALDI-TOF MS and identified 40 spots, which were matched using the MASCOT peptide mass fingerprints (PMF) to entries in the SWISS-PROT database with high confidence. Among the 40 spots, 21 spots were upregulated and 19 spots were downregulated in hMSC-miR-3148 compared to control cells (Fig. 2c, Supplementary Fig. 4, and Supplementary Table 2). Pathway analysis of the identified proteins revealed strong enrichment in pathways associated with cancer development (Fig. 2d).

### miR-3148 overexpression enhances colony formation, cell migration and invasion

**Fig. 3:**
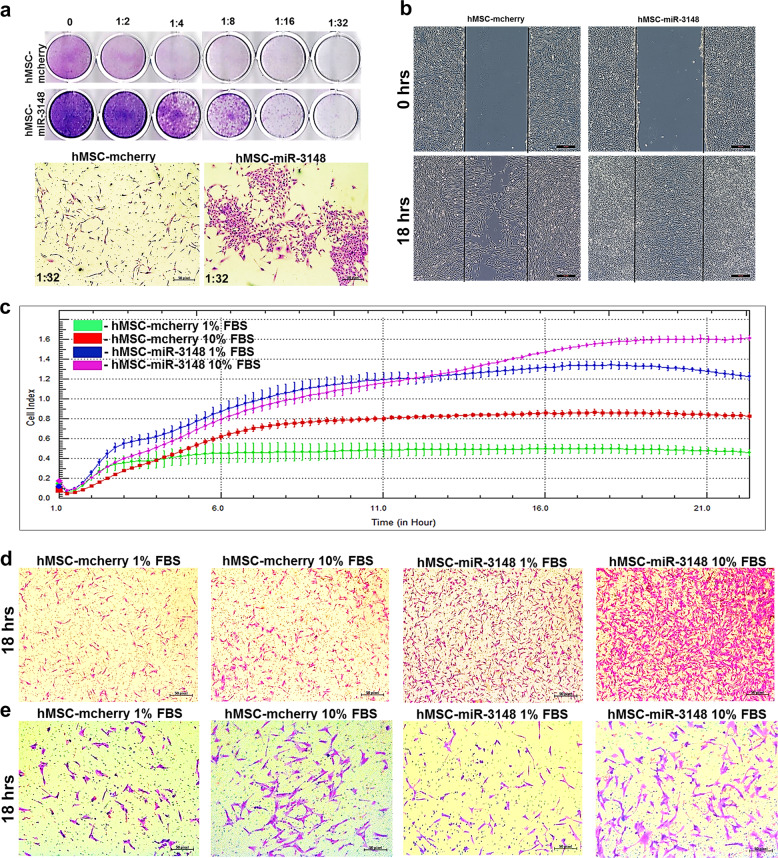
The hMSC-miR-3148 cells exhibited enhanced clonogenicity, migration and invasion potential. **a** Clonogenic (CFU) assay showing enhanced colony forming capability of hMSC-miR-3148 compared to hMSC-mcherry control cells on day 5. Plates were stained using the Diff-Quik staining set. Data are representative of two independent experiments for each condition and representative images of formed colonies (lower panels) were taken at 1:32 dilution. **b** Measurement of hMSC-miR-3148 cell migration using in vitro scratch assay. Images were captures just after the injury (0 h) and at 24 h. XCELLigence real-time cell analysis (RTCA) (**c**) and conventional (**d**) migration assay depicting enhanced migration of hMSC-miR-3148 cells toward 1 and 10% serum condition compared to hMSC-mcherry control cells in a time-dependent manner. **e** Representative images from conventional invasion assay demonstrating enhanced invasion capabilities hMSC-miR-3148 compared to hMSC-mcherry control cells.

Transcriptomic and proteomic data revealed enrichment in cancer-associated functional categories and signaling pathways in the hMSC-miR-3148 model. Therefore, we subsequently investigated the biological effects of forced expression of miR-3148 on hMSC colony formation, migration, and invasion. Data presented in Fig. 3a revealed increased colony formation in hMSC-miR-3148 compared to control cells, which were observed in hMSC-miR-3148 as early as day 5, compared to control cells. Scratch and xCELLigence real-time migration assays revealed significant increase in cell motility and migration of hMSC-miR-3148 (Fig. 3b, c). In addition, Boyden-chamber transwell migration and invasion assay, revealed enhanced migration (Fig. 3d) and invasion (Fig. 3e) of hMSC-miR-3148.

### miR-3148 overexpression enhances cell proliferation and doxorubicin drug resistance

**Fig. 4:**
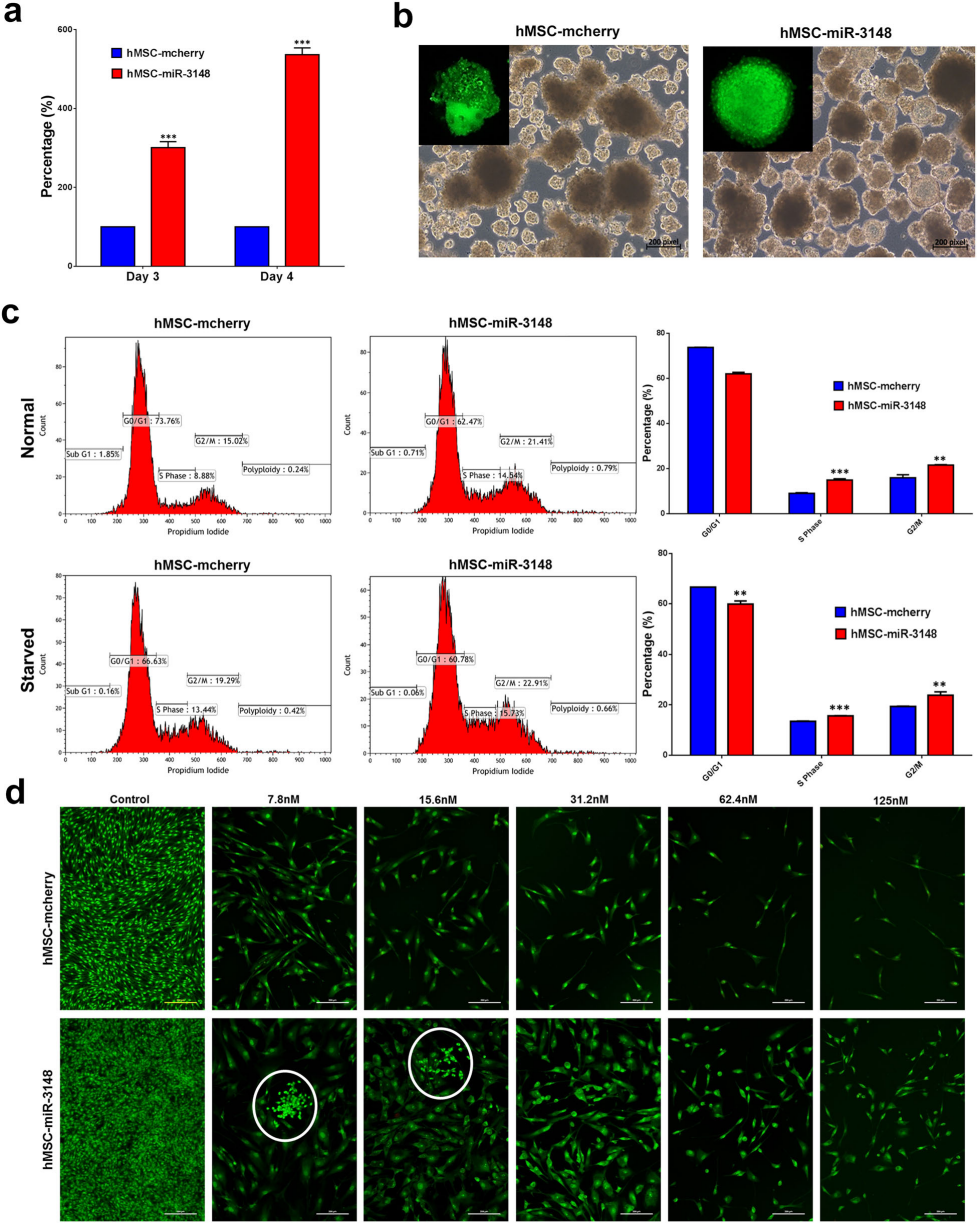
The hMSC-miR-3148 cells exhibited enhanced cell proliferation, sphere formation, and doxorubicin resistance. **a** Cell proliferation measured using alamarblue assay of hMSC-miR-3148 compared to hMSC-mcherry cells on day 3 and day 4. Data are presented as mean percent increase in cell proliferation ± S.E.M., *n* = 30 from two independent experiments, ***p < 0.0001. **b** Representative images depicting sphere formation in hMSC-miR-3148 vs hMSC-mcherry cells on day 10. **c** Flow cytometry cell cycle analysis on hMSC-miR-3148 and hMSC-mcherry under normal (upper panels) and serum starvation (lower panels) conditions. The proportion of cells in the G0-G1, S, and G2-M phases are indicated on each plot. Quantitative analysis of cell cycle distribution in hMSC-miR-3148 and hMSC-mcherry is presented as bar diagram. Data are presented as mean ± S.E.M., *n* = 3, **p < 0.005; ***p < 0.0005. **d** Representative fluorescence images of hMSC-miR-3148 and hMSC-mcherry control cells (±different concentration (7.8–125 nM) doxorubicin). Cells were stained with acridine orange/ethidium bromide to detect apoptotic and necrotic cells. White circles indicate resistant colonies.

hMSC-miR-3148 cells exhibited increased cell proliferation (Fig. 4a), formed a greater number of spheroids when cultured under low-adherence cell culture conditions (Fig. 4b). Notably, the spheroids formed by hMSC-miR-3148 were more compact whereas control cells-derived spheroids had irregular forms (Fig. 4b, zoomed). Cell cycle analysis revealed significant decrease in G0/G1 and significant increase in S and G2-M phases of the cell cycle in hMSC-miR-3148 compared to controls (Fig. 4c). Finally, hMSC-miR-3148 and control cells were exposed to doxorubicin, a commonly used chemotherapy in the treatment of human sarcomas^14^. hMSC-miR-3148 exhibited increased resistance to doxorubicin concentration ranging from 7.8 to 125 nM (Fig. 4d) and formation of doxorubicin-resistant colonies was notable at 7.8 and 15.6 nM.

### miR-3148 overexpression promotes sarcoma-like tumor formation in vivo

**Fig. 5:**
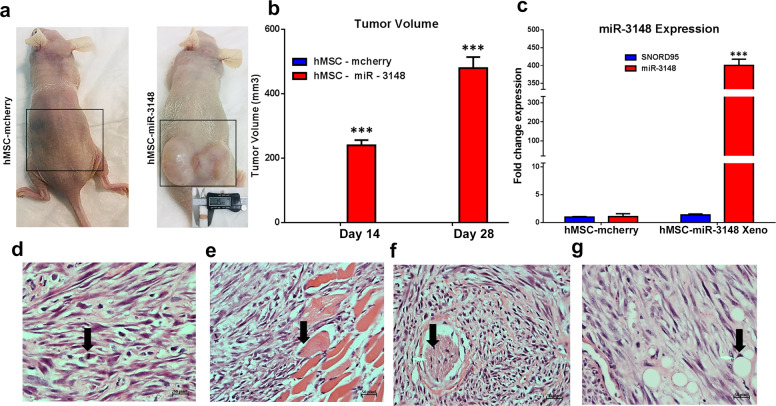
The hMSC-miR-3148 cells exhibited enhanced tumor formation in vivo. Both hMSC-miR-3148 and hMSC-mcherry control cells were injected subcutaneously in nude mice. Representative images of tumor formed in each experimental group on day 28 is shown in (**a**). **b** Tumor size (log scale) in each experimental group on day 14 and day 28 is presented as bar chart. Data are presented as mean tumor volume ± S.E.M., *n* = 4; ***p < 0.0005. **c** qRT-PCR quantification of hsa-miR-3148 expression in hMSC-miR-3148 Xenograft tissue compared to mcherry control cells. Data are representative of three experiment and are presented as mean ± S.D., *n* = 3. **d**–**g** Representative histopathological examination of hMSC-miR-3148 xenograft tumors using haematoxylin and eosin stain. Arrows indicate mitotic figures (**d**), muscle invasion (**e**), nerve surrounding (**f**), and adipocyte formation (**g**).

Our in vitro data demonstrated enhanced cell proliferation, colony formation, and migration in hMSC-miR-3148 cells. Therefore, to examine the ability of hMSC-miR-3148 to form tumors in vivo, we implanted hMSC-miR-3148 and control cells loaded into Matrigel® matrix, subcutaneously into immune deficient nude mice. Concordant with the in vitro data, hMSC-miR-3148 cells formed tumors, which were not detected in control cells (Fig. 5a, b). We observed sustained high expression of miR-3148 in the hMSC-miR-3148 xenografts (Fig. 5c). Histological examination of the tumor tissue revealed a sarcoma-like tumor tissue with numerous spindle and pleomorphic cells and with mitotic figures (Fig. 5d). The hMSC-miR-3148 tumors invaded skeletal muscles (Fig. 5e) and surrounding nerves (Fig. 5f). Interestingly, tumor tissue contained adipocytes suggesting a degree of adipocyte differentiation (Fig. 5g).

### miR-3148 regulates TGFβ signaling pathways through direct targeting of SMAD2

**Fig. 6:**
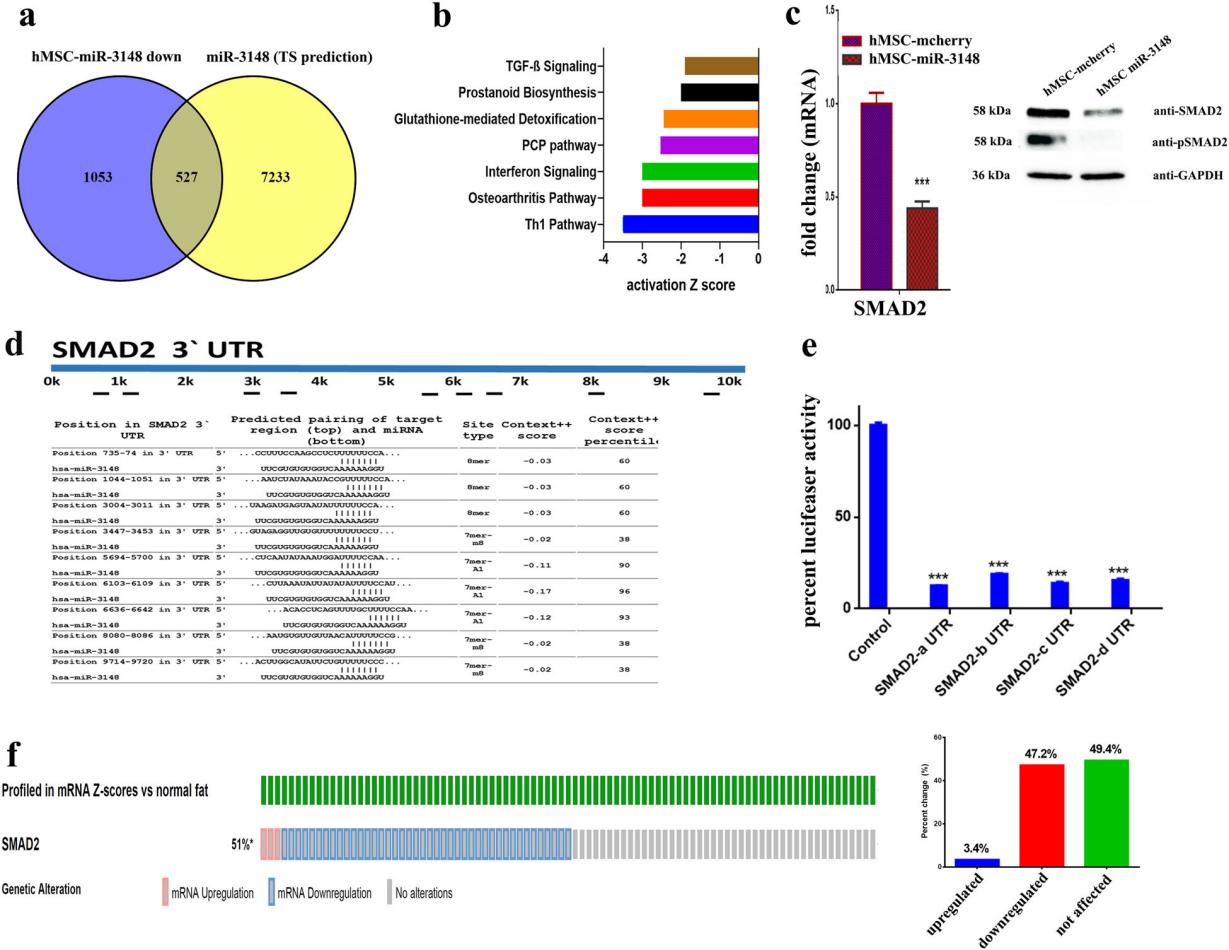
miR-3148 regulates TGFβ signaling through direct targeting of SMAD2. **a** Venn diagram depicting the overlap between the downregulated mRNA transcripts in hMSC-miR-3148 cells and in silico predicted gene targets for miR-3148 (based on TargetScan algorithm). **b** Significantly altered canonical pathways revealed by IPA on the downregulated transcripts in hMSC-miR-3148 cells. **c** qRT-PCR and immunoblotting showing the downregulation of SMAD2 and pSMAD2 proteins in hMSC-miR-3148 compared to control cells. **d** Illustration of the alignment of miR-3148 and SMAD2 3′ UTR region using TargetScan algorithm indicated nine potential binding sites for miR-3148. **e** Luciferase-based reporter assay illustrating the direct interaction between miR-3148 and SMAD2 3′UTR. Data are presented as mean ± S.E.M., *n* = 5 from two experiments. The two-tailed *t* test was used to compare different treatment groups. **f** Oncoprint and bar chart depicting altered SMAD2 mRNA expression in soft tissue sarcoma from the SKCC/BI soft tissue sarcoma dataset.

In order to understand the molecular mechanism of miR-3148, we compared the list of downregulated transcripts in hMSC-miR-3148 cells to in silico predicted gene targets using the Targetscan database^15^ and targetscan algorithm^16^. We identified 527 downregulated transcripts in hMSC-miR-3148 (Fig. 6a) and pathway analysis revealed significant inhibition of a number of canonical pathways related to osteogenesis, including TGFβ pathway (Fig. 6b). Interestingly, we observed several members of the SMAD family, which are TGFβ downstream effectors, to be significantly downregulated: SMAD2: −4.1 FC, SMAD6: −5.2 FC, and SMAD9: −4.4 FC (Supplementary Table 1). The expression of SMAD2 was subsequently validated using qRT-PCR and immunoblotting, given its key role in mediating the TGFβ signaling pathway. As seen in Fig. 6c, the expression and phosphorylation of SMAD2 were downregulated in hMSC-miR-3148. In silico analysis revealed nine potential binding sites for miR-3148 in SMAD2 3′ UTR (Fig. 6d). Luciferase-based reporter assay corroborated our findings and validated SMAD2 as bone fide target for miR-3148 (Fig. 6e).

**Fig. 7:**
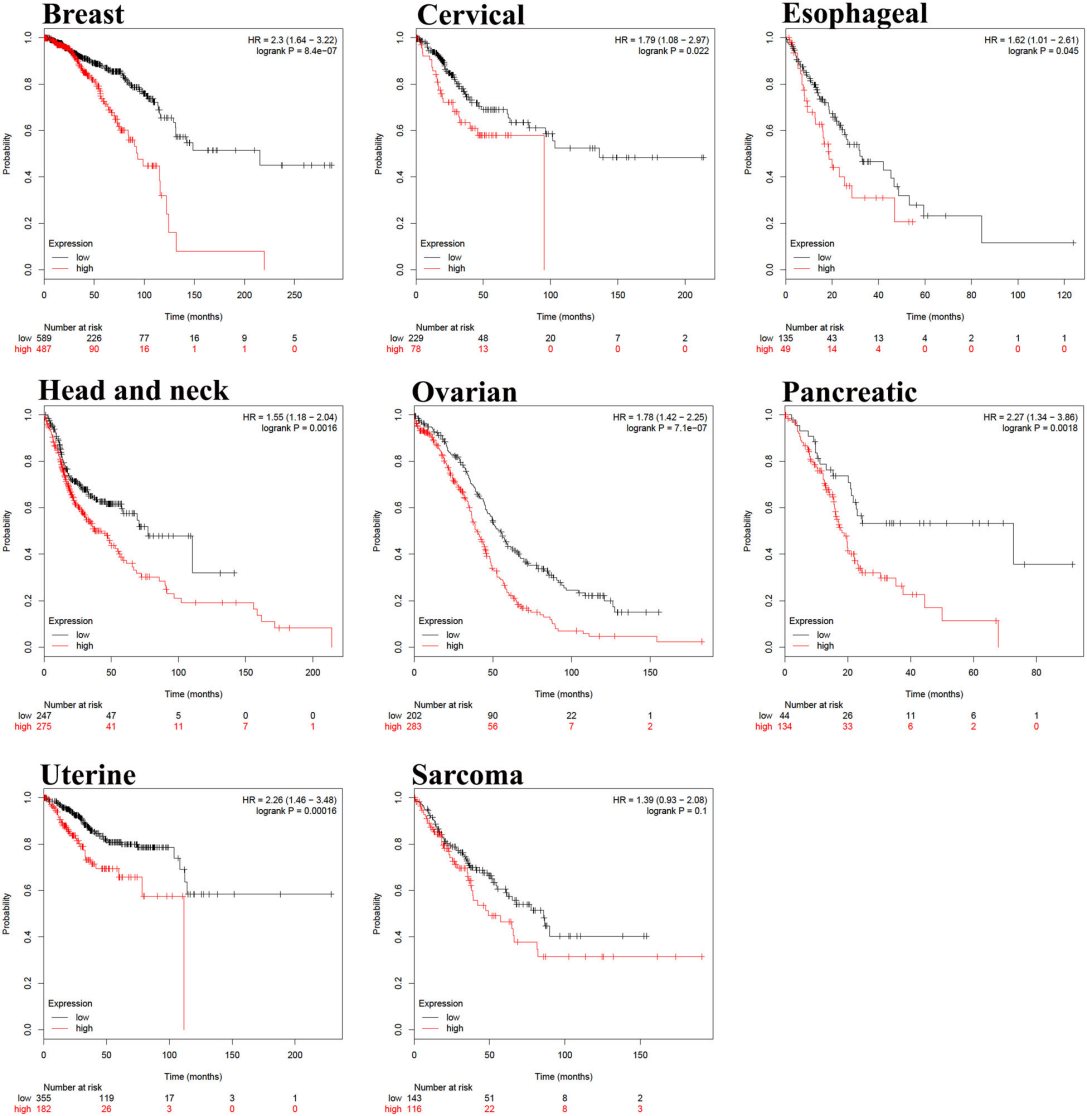
Higher expression of miR-3148 is unfavorable prognostic factor in multiple cancer types. Kaplan–Meier overall survival analysis for miR-3148 expression in breast, cervical, esophageal, head and neck, ovarian, pancreatic, uterine cancer and sarcoma patients. Analyses were conducted using Kaplan–Meier plotter available at http://kmplot.com/analysis/index.php?p=background. Survival curve comparison was performed using the log-rank test.

To highlight the clinical relevance of our findings, we examined the expression of SDMAD2 in the Memorial Sloan Kettering Cancer Center and Broad Institute (SKCC/BI) soft tissue sarcoma dataset, which revealed significant downregulation of SMAD2 in 47% of sarcoma patients in these cohorts (Fig. 6f). In addition, higher expression of hsa-miR-3148 was associated with lower overall survival in multiple human cancer types (breast, cervical, esophageal, head and neck, ovarian, pancreatic, and uterine) and sarcoma (Fig. 7).

## Discussion

miRNAs orchestrate complex regulatory networks across various biological systems. Each miRNA can regulate dozens of mRNA transcripts; and conversely, a single mRNA transcript can be regulated by several miRNAs. Interestingly, a single miRNA can exert multiple effects in various biological system. Dysregulated miRNA expression has been implicated in human diseases, including diabetes, cardiovascular, kidney diseases and cancer^17–19^. For instance, the let-7 family of miRNAs regulates stem cell differentiation and predominantly exert a tumor suppressor effects^20,21^. Similarly, we have previously reported that miR-320 family plays a role in hMSC osteoblastic and adipocytic differentiation and to suppress colorectal cancer proliferation and migration^11,12^. In the context of carcinogenesis, miRNA can be classified as oncomiRs or as tumor suppressor miRNAs. OncomiRs mediate their effects through downregulation of tumor suppressor genes, leading to abnormal cell functions and tumor development and progression^22^. For example, the miR-21 oncomiR is overexpressed in many human cancers, including, thyroid, breast, lung, CRC, pancreas, liver, gastric, cervical, skin, glioblastoma, and hematological malignancies^23,24^.

We observed that overexpression of miR-3148 in hMSC led to enhanced cell proliferation, induced a transformed cellular phenotype as well as formation of sarcoma-like tumors when implanting the cells in vivo. We have also observed that miR-3148 targets TGFβ signaling pathway. The role of TGFβ and BMPs is well documented during bone formation, which signals through both canonical SMAD-dependent and noncanonical SMAD-independent signal transduction pathways^25^. In the context of tumorigenesis, TGFβ pathway exerts opposing role either as promoter or as suppressor of tumor development and progression^26^. Several studies highlighted an important role for TGFβ in regulating cell proliferation and promoting apoptosis in normal and transformed cells^27^. In breast and colon cancers, TGFβ signaling exerts antineoplastic effect during early stages, but eventually it promotes carcinogenesis through mediating epithelial mesenchymal transition (EMT)^28^. Interestingly, disruption of TGFβ signaling in the prostate of SV40 large T antigen transgenic animals, did not affect the size of neoplastic tumors, but instead it promoted tumor metastasis^29^. Also, disruption of SMAD2 accelerates malignant progression of intestinal tumors in apc knockout mice, although the total number of intestinal tumors in those animals was not affected^30^. Taken together, the current studies suggest that disruption of TGFβ accelerates tumorigenesis, while the loss of TGFβ signaling is not sufficient to trigger malignant transformation. Our data are in agreement with the aforementioned reports where we observed miR-3148 to promote malignant transformation of the telomerized-hMSC cells, while forced expression of miR-3148 on its own has no significant effects on the growth of primary skin derived stromal cells (Supplementary Fig. 4). Telomerized-hMSC model employed in current study has normal karyotype, but exhibited deletion of the Ink4a/ARF locus and therefore is more prone to malignant transformation^31^, which provides a plausible explanation for the observed data of differential effects of miR-3148 on telomerized-hMSC compared to primary stromal cells. It also suggests that inactivation of TGFβ via miR-3148/SMAD2 axis is a “secondary hit” accelerating malignant transformation. In Ewing’s sarcoma (ES), elevated expression of miR-20b was shown to directly target TGFβ receptor II (TGFBR2) leading to elevated MYC expression, hence inhibition of miR-20b reduced ES cell grow, cell cycle progression, and in vivo tumor formation, thus corroborating the important role of loss of TGFβ signaling in promoting ES tumor formation^32^. In addition to the SMAD2/TGFB pathway, miR-3148-hMSC cells exhibited upregulation and downregulation of several onco- and tumor suppressor genes, respectively. The role of other genes in regulating hMSC differentiation and tumorigenesis in the context of miR-3148 remains to be elucidated.

MiR-3148-hMSC cells exhibited enhanced resistance to doxorubicin, a commonly used chemotherapy in the treatment of human sarcomas. In order to provide potential mechanism by which miR-3148 confers doxorubicin resistance, gene expression data from GSE3362 was retrieved and the upregulated and downregulated genes in doxorubicin-resistant sarcoma were identified and were crossed with the list of differentially expressed genes in miR-3148-hMSC cells. Our data revealed common upregulated (ACADVL, RAG1, CYP51A1, PLAT, ITGA5, LOX, MAP4, MMP1, SCG5, STC1, THBS1, ZNF91, TNFRSF25, DDIT3, SLC16A3, SYNGR2, XRCC3, SEMA3A, POLR3G, TFPI2, KLF2, REEP2, TOR4A, STEAP3, APBB1IP, and SLC25A19) and common downregulated (CYP1B1, GJA1, IGFBP2, GSTM3, CD70, AMPH, LIF, MSX1, OAS2, THBS4, TPD52L1, BHLHE40, RUNX3, FGL1, DHRS3, FGR, SLC7A8, CD24, CELSR1, TCAF1, DACT1, PHF10, FEZ1, and C15orf48), Supplementary Fig. 6. For instance, RUNX3 was previously shown to inhibit hepatocellular carcinoma growth in vitro and in vivo in combination with Adriamycin^33^. Concordantly, reduced expression of AMPH1 was previously shown to be associated with breast cancer metastatic, advanced stage, poor clinical outcome, and Paclitaxel plus 5-fluorouracil/ epirubicin/cyclophosphamide treatment resistance^34^. The role of other genes in mediating doxorubicin residence in miR-3148-hMSC warrants further investigation.

A number of studies highlighted various mechanism leading to malignant transformation of hMSCs. For instance, methylation of HIC ZBTB Transcriptional Repressor 1 (HIC1) and Ras Association Domain Family Member 1 (RassF1A) tumor suppressor genes was sufficient to transform bone marrow MSCs into malignant cells endowed with tumor‐initiating capabilities^35^. Alternatively, a number of genetic modification were utilized to transform hMSCs into malignant sarcoma cells. The combination of hTERT/H-RAS and hTERT/H-RAS/SV40-LT has been demonstrated to induce pleomorphic sarcoma cells^36–38^. Transformed hMSCs using combination of hTERT and H-RAS retained their adipocytic and chondrogenic, while osteoblastic differentiation was completely lost^38^. Our current study corroborates these findings since miR-3148 overexpressing hMSC-TERT cells exhibited enhanced adipogenesis and suppressed osteogenesis, suggesting the loss of osteogenic differentiation is a common feature during malignant transformation of hMSC. Interestingly, we found positive correlation between elevated expression of miR-3148 and poor prognosis in several human cancer types, including sarcomas thus highlighting miR-3148 as a possible prognostic biomarker in various human cancers. In support of this hypothesis, recent data reported higher expression of miR-3148 in 3D-spheroids formed using the HCT116 human CRC model^39^. miR-3148-overexpressing HCT116 cells exhibited enhanced tumor formation in vivo, were resistant to hypoxic conditions and exhibited higher sensitivity to MAPKK and ERK inhibitors^39^.

In addition to its possible role in carcinogenesis, a role for miR-3148 in the context of other pathological conditions has been reported. miR-3148 levels were inversely correlated with Toll-like receptor 7 (TLR7) transcript levels in peripheral blood monocytes obtained from systemic lupus erythematosus (SLE) patients^40^. Interestingly, the G allele of rs3853839 in the 3’ UTR of TLR7 has diminished binding capability to miR-3148, which was associated with elevated TLR7 transcript expression and increased risk for SLE, thus implicating miR-3148-TLR7 circuit in SLE pathogenesis^40^. Elevated levels of miR-3148 were also associated with congestive heart failure (CHF) in a cohort of 44 CHF patients and 15 healthy subjects^41^. In addition, downregulation of miR-3148 was observed in chronic pulmonary hypertension^42^.

In conclusion, our data provide novel insight on the role of miR-3148-SMAD2-TGFβ axis during malignant transformation of hMSCs into sarcoma-like cancer and suggest therapeutic approaches targeting miR-3148/SMAD2/TGFβ pathway as plausible therapeutic strategy.

## Materials and methods

### Cell culture and lentiviral transduction

We employed a well-characterized telomerase-transduced adult bone marrow hMSC cell line (hMSC-TERT) that expresses all known markers and possess similar differentiation potential to primary hMSCs in vitro and in vivo^43,44^. For simplicity, this line is referred to as hMSC throughout the paper. Those cells were cultured in Dulbecco’s modified Eagle’s medium (DMEM) supplemented with 4500 mg/l D-glucose, 4 mM L-glutamine, 110 mg/l sodium pyruvate, 10% fetal bovine serum (FBS), 1% penicillin-streptomycin, and nonessential amino acids as previously described^43^.

Lentiviral particles encoding for hsa-miR-3148 (LP-HmiR0781-MR03-0200-S) and control particles were purchased from Genecopoeia (Genecopoeia Inc., Rockville, MD, USA). Transduction was performed as previously described^12^. Briefly, hMSCs were treated with 20 μl of crude lentiviral particles in 500 μl of transduction medium (DMEM + 5% heat-inactivated serum and 1% Pen-Strep (Invitrogen)) with polybrene (8 μg/ml; Sigma, St. Louis, MO, USA). After 24 h, media was removed and cells were selected with puromycin (1 μg/ml, Sigma, St. Louis, MO, USA) for 1 week until stably transduced cells were generated. Transduction efficiency was confirmed using qRT-PCR for miR-3148 expression compared to mcherry-transduced cells.

### Gene expression profiling by microarray

Gene expression profiling was performed as previously described^45^. In brief, total RNA was extracted using the total tissue RNA purification kit from Norgen-Biotek Corp. (Thorold, ON, Canada) and quantified in NanoDrop 2000 spectrophotometer (Thermo Scientific, Wilmington, DE, USA). One-hundred fifty ng of total RNA was labeled and then hybridized to the Agilent Human SurePrint G3 Human GE 8 × 60k microarray chip and subsequently data were analyzed using GeneSpring 13.0 software (Agilent Technologies, Palo Alto, CA, USA). Single experiment pathway analysis tool was used with twofold expression cutoff (*p* < 0.05). Benjamini–Hochberg False Discovery Rate (FDR) method was used for multiple testing corrections.

### Ingenuity pathway and network analysis

Pathway analyses were conducted using Ingenuity pathway (Ingenuity Systems, http://www.ingenuity.com)^46^. Differentially expressed genes exhibiting ≥ 2 ≤ and *p* value < 0.05 were subjected to core analysis using the human database. Enriched networks categories were algorithmically generated based on their connectivity and ranked according to the *Z* score.

### qRT-PCR

The expression levels of the indicated mRNA transcipts were validated using SYBR Green-based qRT-PCR using the applied biosystems ViiA™ 7 real-time PCR system (ThermoFisher Scientific). Primers used in current study are listed in Table 1. Differential expression was calculated using the 2∆CT method and data were plotted as bar diagram.

**Table 1:** List of SYBR green primers used in current study.

No	Name	Sequence
1	AP2	F 5′ TGGTTGATTTTCCATCCCAT
		R 5′ GCCAGGAATTTGACGAAGTC
2	LEP	F 5′ CAGCGGTTGCAAGGCCCAAGA
		R 5′ GGCCAAAGCCACAAGAATCCGC
3	OC	F 5′ GGCAGCGAGGTAGTGAAGAG
		R 5′ CTCACACACCTCCCTCCTG
4	ON	F 5′ GAGGAAACCGAAGAGGAGG
		R 5′ GGGGTGTTGTTCTCATCCAG
5	ALP	F 5′ GGAACTCCTGACCCTTGACC
		R 5′ TCCTGTTCAGCTCGTACTGC
6	RUNX2	F 5′ GTAGATGGACCTCGGGAACC
		R 5′ GAGGCGGTCAGAGAACAAAC
7	β-actin	F 5′ AGCCATGTACGTTGCTA
		R 5′ AGTCCGCCTAGAAGCA

### Protein extraction for mass spectrometry

Proteins were extracted from three biological replicate (~2 × 10^6^ cells) from the hMSC-miR-3148 or hMSC-mcherry cells directly in lysis buffer (0.5 mL, pH 8.8, 30 mM Tris buffer containing 7 M urea, 2 M thiourea, 2% Chaps, 1× protease inhibitor mix). The suspension was shaken for 1 h at room temperature and then sonicated (Microsonicator, Qsonica Sonicators, USA; 30% pulse, two intervals of 1 min each, separated by a 1 min gap in ice). Fifty mM dithiothreitol was then added and the protein extracts were centrifuged (20,000 × *g*, 1 h, 4 °C). The pellet was then removed and the solubilized proteins in the supernatant were precipitated using a 2D clean-up kit according to the manufacturer’s protocol (GE Healthcare, USA).

### Protein labeling with cyanine dyes

The protein pellets were solubilized in labeling buffer (7 M Urea, 2 M Thiourea, 30 mM Tris-HCl, 4% CHAPS, pH 8.5). Insoluble materials were pelleted by centrifugation (12,000 × *g*, room temperature (RT), 5 min), protein concentrations were determined in triplicate using the 2D-Quant kit (GE Healthcare, USA), and the pH of the samples was adjusted to 8.5 using NaOH (100 mM). Proteins were labeled (400 pmol of CyDye™ DIGE Fluor dyes, GE Healthcare, UK) in 1 μl of DMF and then mixed with sample containing 50 μg of protein. Samples were incubated on ice for 30 min in the dark. The labeling reaction was terminated by adding 1 μl of 10 mM lysine. Each sample was covalently labeled with a fluorophore, either Cy3 or Cy5. A mixture of equal amounts of protein isolated from each and every sample in the experiment was labeled with Cy2 and used as internal standard.

### Two dimensional gel electrophoresis (2D-DIGE)

First dimension analytical gel electrophoresis was performed as follows. Six Immobiline Dry Strips (24 cm, pH 3–11; GE Healthcare, Sweden) were passively re-hydrated (30 V, 12 h). This was followed by isoelectric focusing using an Ettan IPGphor IEF unit (GE Healthcare, Sweden). Focusing was performed at 20 °C, at 50 μA per strip, according to the following step and hold sequence: i.e., (1) 500 V for 1 h, (2) 1000 V for 1 h, (3) 8000 V for 3 h, (4) 45,000 V for 1 h. After the first dimension, the strips were equilibrated and separated on 12.5% (SDS-PAGE) gels using an Ettan Dalt Six device (GE Healthcare, Sweden). The gels were scanned with a Typhoon 9400 scanner (GE Healthcare) using appropriate wavelengths and filters for Cy2, Cy3, and Cy5 dyes. Total protein (1 mg) was obtained from a pool of equal protein amounts of each sample. This was denatured in lysis buffer, then mixed in a rehydration buffer. Gels were then stained by Colloidal Coomassie Blue staining (Supplementary Fig. 4).

### Protein Identification by MALDI-TOF MS

The spots from Coomassie-stained gels were excised manually, washed, and digested according to a previously published protocol^47^. The mixture of tryptic peptides (1 µL), derived from each protein, was spotted onto a MALDI target (384 anchorchip MTP 800 µm Anchorchip; BrukerDaltonik, Germany) together with 0.8 μL of matrix (10 mg α-cyano-4-hydroxycinnamic acid (CHCA) in 1 μL of 30% CH3CN and 0.1% aqueous CF3COOH) and then left to dry (RT) before MS analysis. Spectra were acquired using a MALDI-TOF MS (UltraFlexTrem, Bruker Daltonics) in the positive mode with target voltage of 25 kV and pulsed ion extraction voltage of 20 kV. The reflector voltage was set to 21 kV and detector voltage to 17 kV. PMF were calibrated against a standard (Peptide Calibration Standard II, Bruker Daltonics). The PMF were processed using the Flex AnalysisTM software (version 2.4, Bruker Daltonics). The MS data were interpreted using BioTools v3.2 (Bruker Daltonics), together with the MASCOT search algorithm (version 2.0.04 updated 09/05/2017; Matrix Science Ltd., UK). MASCOT search parameters were set as follows: fixed propionamide modification of cysteine, oxidation of methionine as variable modification, one missed cleavage site (such as in the case of incomplete trypsin hydrolysis), and a mass tolerance of 100 ppm. Identified proteins were accepted as correct if they showed a MASCOT score > 56 and *p* < 0.05. Not all spots of interest could be identified because some proteins were low in abundance and did not yield a sufficiently intense mass of fingerprints; other spots were mixtures of multiple proteins.

### Image acquisition and statistical analysis

DIGE images were analyzed using Progenesis Same Spots v3.3 software (Nonlinear Dynamics Ltd., UK). First, images were aligned. Prominent spots were used to manually assign; 45 vectors to digitized images within each gel and then the automatic vector tool was used to add additional vectors (390 total vectors), which were manually revised and edited for correction if necessary. These vectors were used to warp and align gel images with a reference image of one internal standard across and within each gel. Gel groups were established according to the experimental design and spot normalized volume (NV) was used to select statistically significant spots. The software calculated the NV of each spot on each gel from Cy3 (or Cy5) to Cy2 spot volume ratio. The software performs log transformation of the spot volumes to generate normally distributed data. Log normalized volume (LNV) was used to quantify differential expression. Independent direct comparisons were made between hMSC-miR-3148 and hMSC-mcherry cells and fold differences and *p* values were calculated using one-way ANOVA. All spots were pre-filtered and manually checked before applying the statistical criteria (ANOVA-test, *p* ≤ 0.05 and fold ≥ 1.5). Normalized spot volumes, instead of spot intensities, were used in statistical processing. Only those spots that fulfilled the abovementioned statistical criteria were submitted for MS analysis.

### Protein network Pathway analysis

Pathway analysis was carried out by importing the quantitative data into the IPA software (Ingenuity® Systems, http://www.ingenuity.com). This software aids in determining the functions and pathways that are most strongly associated with the protein list by overlaying the experimental expression data on networks constructed from published interactions.

### Cell differentiation

Adipocytic and Osteoblastic differentiation were performed as previously described^43,48^. In brief, cells were cultured in basal medium till 70–80% confluence. Osteogenic induction medium consisting of DMEM containing 10% FBS, 1% P/S, 50 μg/mL L-ascorbic acid (Wako Chemicals GmbH, Neuss, Germany), 10 mM β-glycerophosphate (Sigma), and 10 nM calcitriol[(1α,25-dihydroxy vitamin D3) (sigma)], 10 nM dexamethasone (Sigma) was added. Adipogenic-induction mixture containing 10% FBS, 10% Horse Serum (Sigma), 1% P/S, 100 nM dexamethasone, 0.45 mM isobutyl methyl xanthine [Sigma], 3 μg/mL insulin (Sigma), and 1 μM Rosiglitazone [(BRL49653) (Novo Nordisk, Bagsvaerd, Denmark)] were added to adipogenic cultures. Both induction media were replaced every 3 days.

### Cytochemical assays

Cytochemical analyses were performed to confirm the adipocyte and osteoblast lineage differentiation of hMSC as previously described^48^. In brief, all cytochemical images were taken using Carl Zeiss—Axio observer1 equipped with digital camera (Axiocam MRc5, Göttingen, Germany).

### Oil red-O staining for adipocytes

Cells were washed in PBS, fixed in 4% formaldehyde and stained for 1 h at RT with filtered Oil red-O staining solution (prepared by dissolving 0.5 g Oil red-O powder in 60% isopropanol). Cells were rinsed with water and then photographed.

### Nile red staining of oil filled droplets

Cells were grown in flat bottom 96-well black microplates (Corning, NY, USA) and after washing, cells were stained with nile red (5 μg/ml in PBS), followed by an incubation for 10 min at RT then washed twice with PBS. Fluorescent signal was measured using SpectraMax/M5 fluorescence spectrophotometer plate reader (Molecular Devices Co., Sunnyvale, CA, USA) using bottom well-scan mode where nine readings were taken per well using Excitation (485 nm) and Emission (572 nm) wavelength.

### Alkaline phosphatase activity (ALP) staining and quantification

We used the BioVision ALP activity colorimetric assay kit (BioVision, Inc., Milpitas, CA, USA) to quantify ALP activity. Cells were cultured in 6-well (for image analysis) and 96-well plates under normal or osteogenic induction conditions, on day 10 cells were washed with PBS and were fixed in 3.7% formaldehyde + 90% ethanol for 30 s at RT. Subsequently, fixative was removed and 50 μl of pNPP (p-nitrophenyl phosphate) solution was added and incubated for 1 h in the dark at RT. Reaction was subsequently stopped by adding 20 μl stop solution then measured and photographed.

### Alizarin Red S (ALZ) staining for mineralized matrix formation

Cells were differentiated and stained on day 21. Twelve well plates were washed with PBS and then fixed with 4% paraformaldehyde for 15 min at RT. Subsequently, cells were washed in distilled water and stained with 2% Alizarin Red S Staining Kit (ScienceCell, Research Laboratories) for 30 min at RT. Subsequently, the dye was washed off with water then plates were photographed and staining intensity was quantified.

### Cell proliferation

The proliferation of hMSC-mcherry and hMSC-miR-3148 cells was determined using alamarBlue assay as previously described^12^. Briefly, 5000 cells were cultured in a 96-well plate and cell proliferation was measured at the indicated time points by adding 1:10 volume of the alamarBlue assay reagent and measuring fluorescence excitation and emission wavelengths of 530 nm and 590 nm, respectively).

### Clonogenic assay

The colony forming ability was determined using clonogenic assay as previously described^12^. Briefly, cells were seeded in 12-well plates at different dilution (1:2–1:32). Primary seeding density was 0.015 × 10^6^ cells per well, and incubated at 37 °C under 5% CO2 for 5 days. The plates were then washed and stained with Diff-Quik stain set (Siemens Healthcare Diagnostics), and subsequently scanned colonies were observed under microscope.

### Sphere formation assay

Multicellular tumor spheroids were produced in 60 mm low cell binding dishes (Nunc; ThermoFisher Scientific). The hMSC-mcherry and hMSC-miR-3148 cells were trypsinized from monolayers and transferred to the dishes. The formation of tumor spheroids was performed with 10,000 cells. On day 10, established spheroids were analyzed and stained with acridine orange and ethidium bromide.

### Cell cycle analysis by flow cytometry

Cell cycle analysis was performed as described earlier^48^, briefly, cell pellets were washed in PBS and re-suspended and stained in 500 μl PBS supplemented 100 μg/ml RNAse A (Sigma, St. Louis, MO, USA) and 50 μg/ml of Propidium Iodine and were analyzed by Navious flow cytometer (Beckman Coulter, Miami, Florida, USA). Staining was detected in the fluorescence channel (FL3) and the data were analyzed using Kaluza software (Beckman Coulter).

### Scratch assay

For the assessment of cell migration, confluent cells were maintained in standard medium and subsequently, scratches were made using a plastic micropipette tip (yellow tip; 20–200 μL). After washing, the medium was replaced with fresh medium. Photographs of the wounded area were taken immediately (0 h) and after 20 h using phase-contrast microscopy.

### Migration assay

Conventional and real-time measurement of cell migration was carried out as described earlier^12^. In brief, real time migration was executed using the xCELLigence RTCA DP device (ACEA Biosciences, San Diego, CA). Cells were starved for 24 h in 1% serum media, followed by seeding 0.08 × 10^6^ cells per well in 16-well microelectronic sensor plate, two chamber transwell plates (CIM-plate insert; ACEA Bioscience) containing the respective serum conditions. Media containing 10% serum (chemo-attractant) and 1% serum (negative control) were added to the bottom wells. Similarly, for conventional migration, the BD transwell migration system with 8μ pore size was utilized. Inserts were placed in a 24-well plate, and 0.1 × 10^6^ cells in 1% serum were added to the top of the chamber, and 10% serum added to the bottom of the chamber. Eighteen hours later, inserts were fixed and stained with SIEMENNS DIFF-QUICK stain set (Siemens Healthcare Diagnostics).

### Invasion assay

For invasion assay, we followed similar set up as in the migration assay, in addition, extracellular matrix (ECM) material—Matrigel was added on top of the transwell membrane. Matrigel was thawed on ice and then 50 µl was added to a 24-well transwell insert and was allowed to solidify at 37 °C for 30 min to form a thin gel layer. Cells were added on top of the Matrigel coating to simulate invasion through the ECM.

### Acridine orange and ethidium bromide staining (AO/EtBr)

AO/EtBr stainig was performed as previously described;^12^ briefly, after doxorubicin drug treatment, both hMSC-mcherry and hMSC-miR-3148 cells were stained with dual fluorescent staining solution (1 μl) containing 100 μg/ml AO and 100 μg/ml EtBr (Sigma, St. Louis, MO). Cells were washed twice with PBS and were gently mixed with AO/EtBr (1:100) dye solution for 1 min. The cells were then observed and photographed using a Nikon Eclipse Ti fluorescence microscope. Cells cultured without drug were considered as experiment control.

### Luciferase reporter assay

Luciferase reporter assay was performed as previously described^12^. Briefly, HEK293 cells were transfected with complexes containing control or SMAD2 UTR plasmids (100 ng), pre-miR control or pre-miR-3148 (50 nM) mixed with lipofectamine 2000 (Part No: 52758; Invitrogen) in Opti-MEM (11058-021; Gibco, Carlsbad, CA, USA). Luciferase activity was measured using the Secrete-Pair™ Dual Luminescence assay kit (Secrete Gaussia luciferase (GLU) and Secreted Alkaline Phosphatase (SEAP); GeneCopeia Inc., USA) according to the manufacturer’s instructions luminescence was measured in SpectraMax M5 (Molecular Devices; USA) luminescence reader. The ratio of luminescence intensities of the GLU over SEAP was calculated and normalized to controls.

### Western blot analysis

SMAD2 and pSMAD2 protein expression was analyzed by western blot as previously described^48^. Briefly, cell lysate samples containing 30 µg of total protein were separated and blotted using the Bio-Rad V3 Western Workflow system according to the manufacturer’s recommendation. Immunoblotting was performed using Phospho-SMAD2 (Ser465/Ser467), cat# E8F3R Rabbit mAb and anti-SMAD2, cat# D43B4 XP® Rabbit mAb, cell signaling technology (Danvers, MA, USA). The primary antibody was incubated overnight at 4 °C. Horseradish peroxidase (HRP)-conjugated goat anti-rabbit was used as the secondary antibody, whereas HRP-conjugated anti-GAPDH (glyceraldehyde-3-phosphate dehydrogenase) antibody (1:1000; Invitrogen) was used as a loading control. Chemiluminescent detection was performed using WesternSure Chemiluminescent Substrate (LI-COR, Lincoln, NE, USA).

### In vivo xenograft assay in nude mice

In vivo tumor formation was carried out as previously described^49^. Briefly, 6–8-week-old male immunodeficient nude mice were utilized in the xenograft experiments. Two million of hMSC-mcherry or hMSC-miR-3148 cells were mixed (1:1) with Matrigel (BD Biosciences, San Jose, CA, USA) and subcutaneously injected into both flanks of nude mice. Tumor mass was measured on day 14 and 28 using a caliper, and tumor volume was calculated using the formula: volume = (tumor length × width × width)/2 as described before^50^. At the end of the experiment, tumors were excised, fixed in 10% buffered formalin, embedded in paraffin, and sectioned on a microtome. Sections were then stained with hematoxylin/eosin and anti-Pan Keratin (AE1/AE3/PCK26).

### **Ethical approval**

Animal experiments received the appropriate institutional ethical from the Animal Care Committees of King Saud University (No. KSU-SE-18-2).

### Immunohistochemistry

Immunohistochemical (IHC) staining protocol was performed as previously described^49^. Four-micron-thick sections were stained using hematoxylin and eosin (H & E) using the IHC staining protocol with fully automated IHC/ISH staining instrument (Ventana Medical system Inc, Tucson, Arizona, USA).

### SMAD2 expression in soft tissue sarcoma and miR-3148 pan-cancer survival analysis

The expression of SMAD2 was interrogated in the (SKCC/BI soft tissue sarcoma dataset as described before^51^. Prognostic value of miR-3148 in breast, cervical, esophageal, head and neck, ovarian, pancreatic, and uterine cancer and sarcoma was investigated as described before^52^.

### Statistical analysis

Statistical analyses and graph generation were performed using Microsoft Excel 2010 and GraphPad Prism 6.0 software (GraphPad, San Diego, CA, USA). *p* values were calculated using the two-tailed *t* test. *p* value < 0.05 was considered significant.

## Supplementary information

Supplementary figure 1

Supplementary figure 2

Supplementary figure 3

Supplementary figure 4

Supplementary figure 5

Supplementary figure 6

Supplementary figure and table legends

Supplementary Table 1

Supplementary Table 2
