# Supplementary figures and images for: MicroRNA-3148 acts as molecular switch promoting malignant transformation and adipocytic differentiation of immortalized human bone marrow stromal cells via direct targeting of the SMAD2/TGFβ pathway
Source: Cell Death Discov. 2020 Sep 1;6:79. doi: 10.1038/s41420-020-00312-z (PMC7462980; doi:10.1038/s41420-020-00312-z)

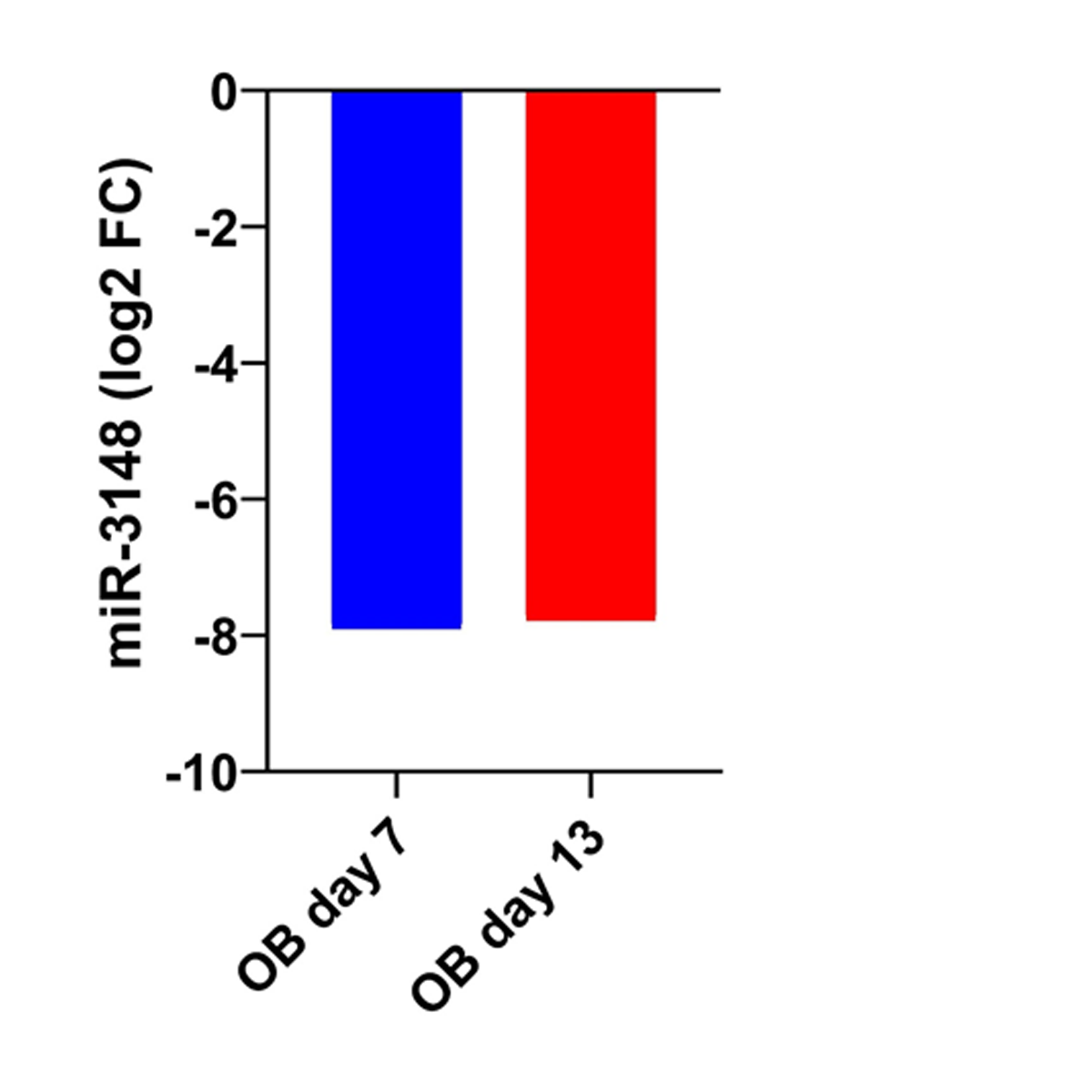

Supplement: Supplementary file 1 — Supplementary figure 1 [file 41420_2020_312_MOESM1_ESM.tif]

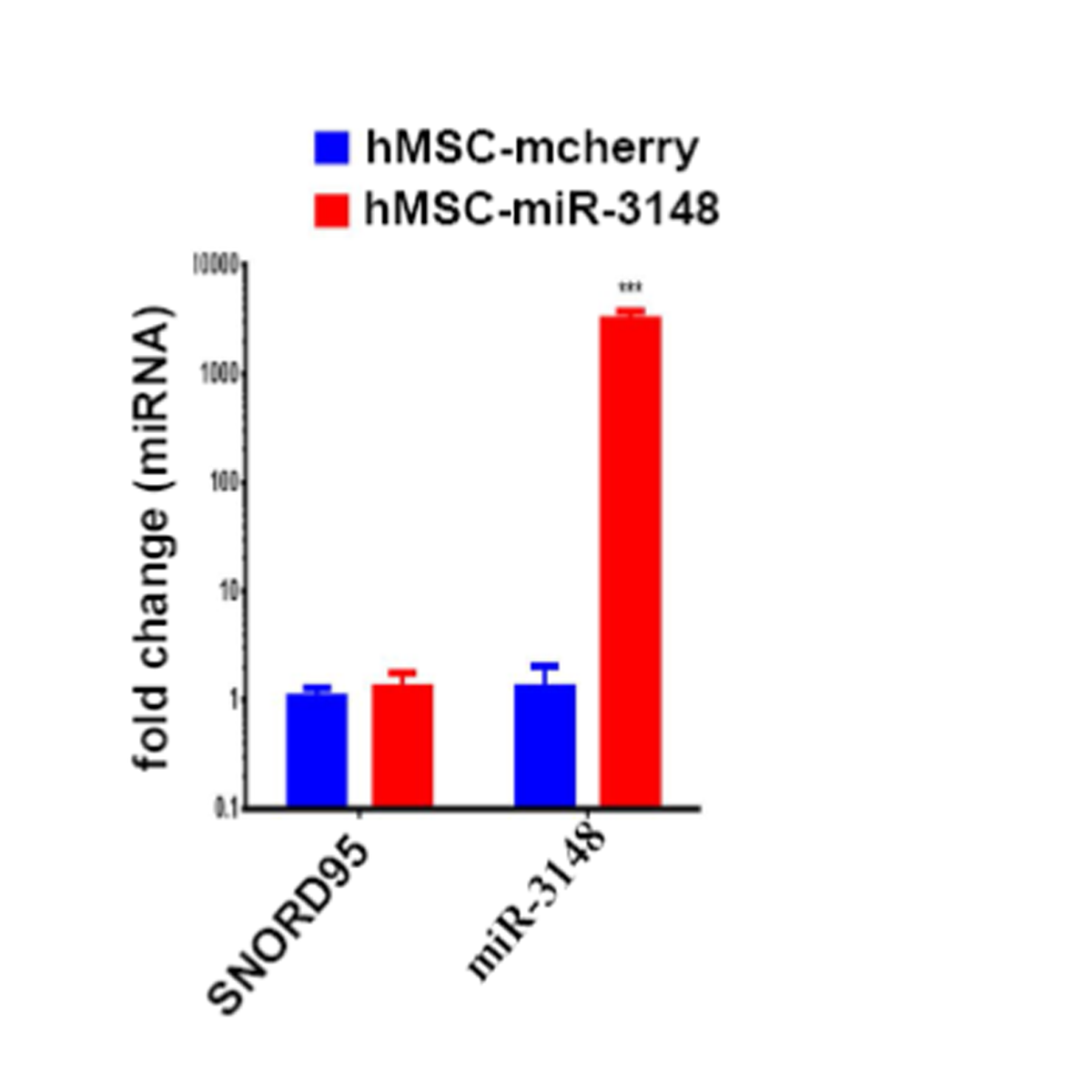

Supplement: Supplementary file 2 — Supplementary figure 2 [file 41420_2020_312_MOESM2_ESM.tif]

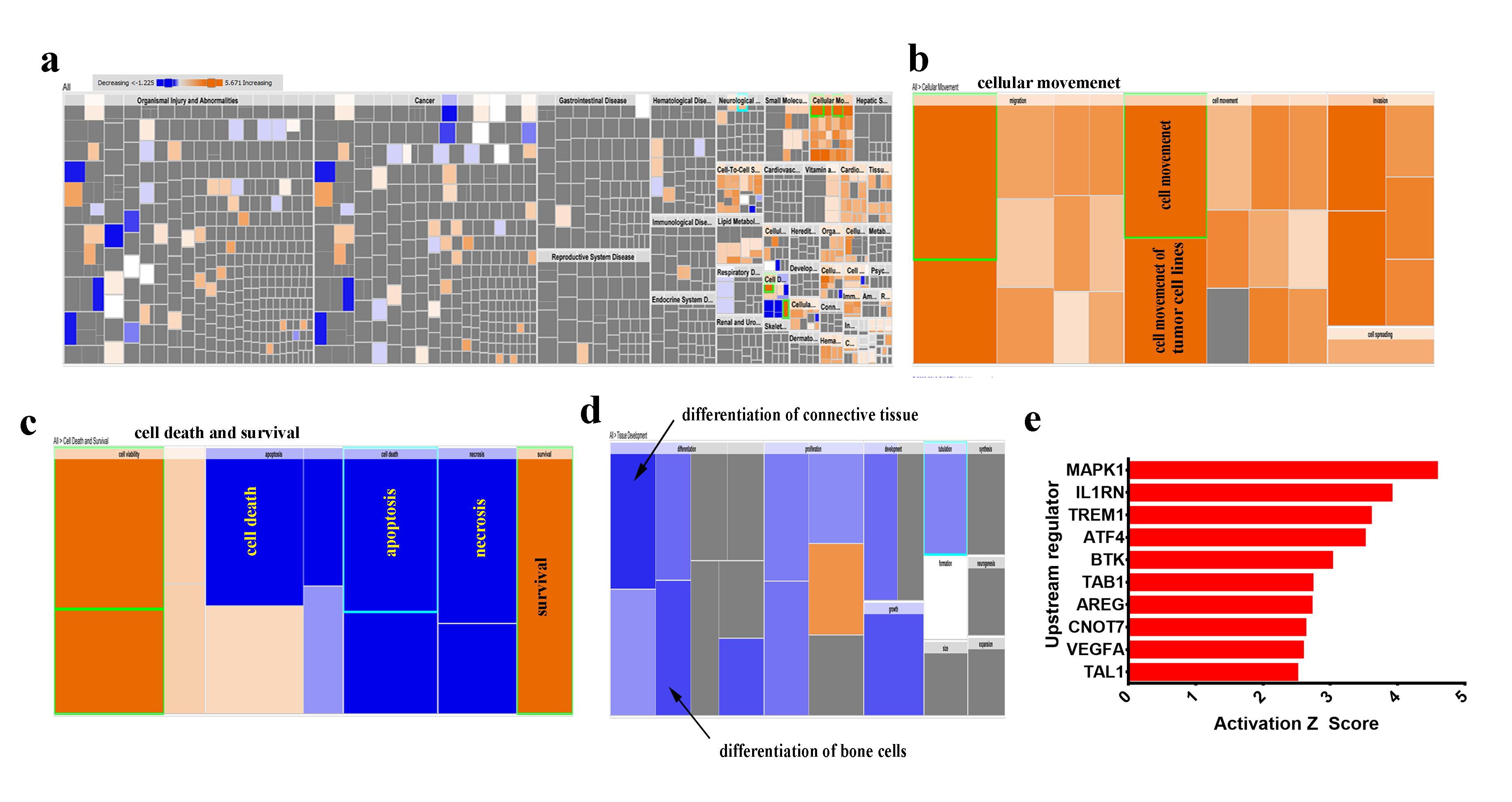

Supplement: Supplementary file 3 — Supplementary figure 3 [file 41420_2020_312_MOESM3_ESM.tif]

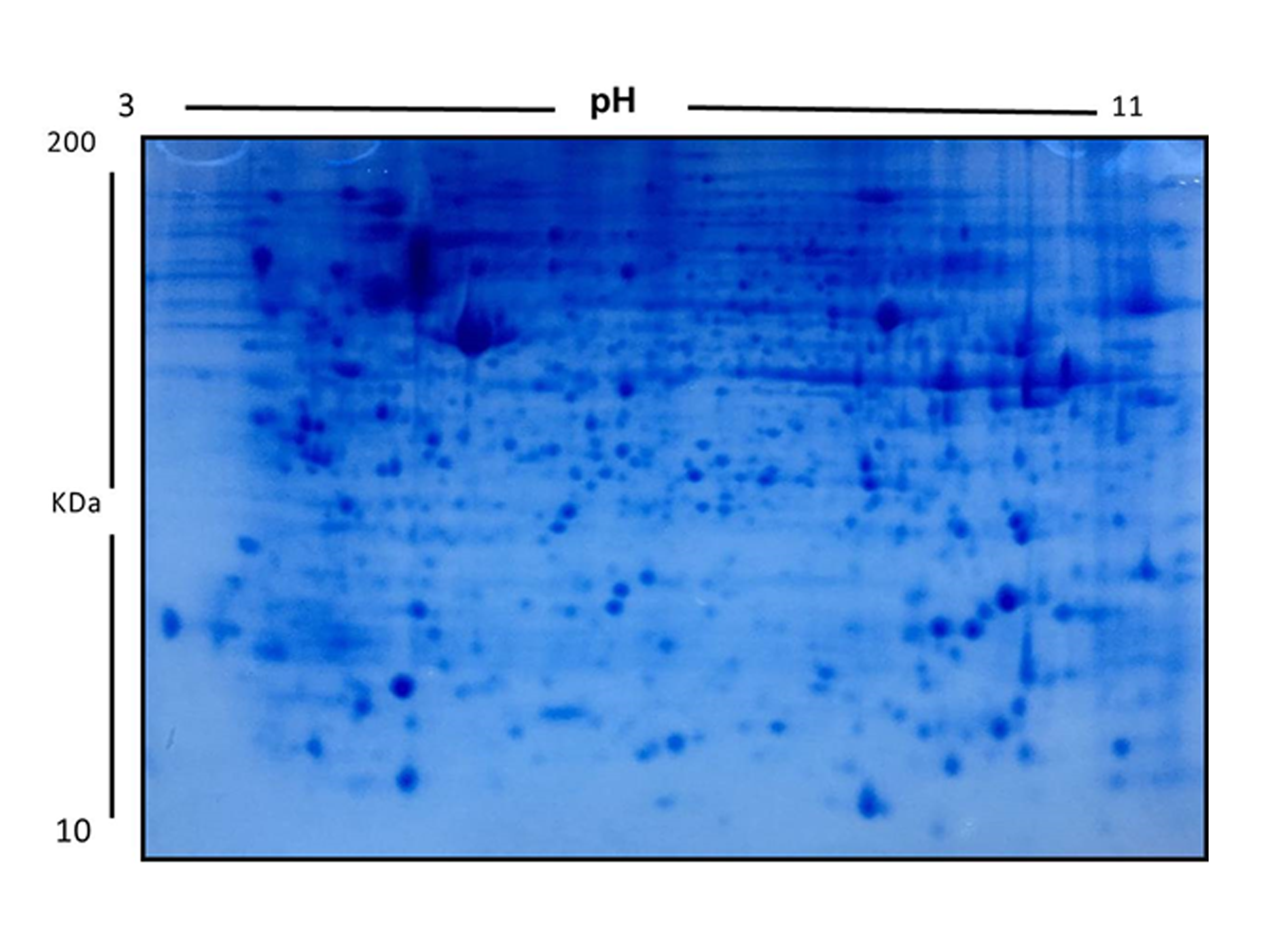

Supplement: Supplementary file 4 — Supplementary figure 4 [file 41420_2020_312_MOESM4_ESM.tif]

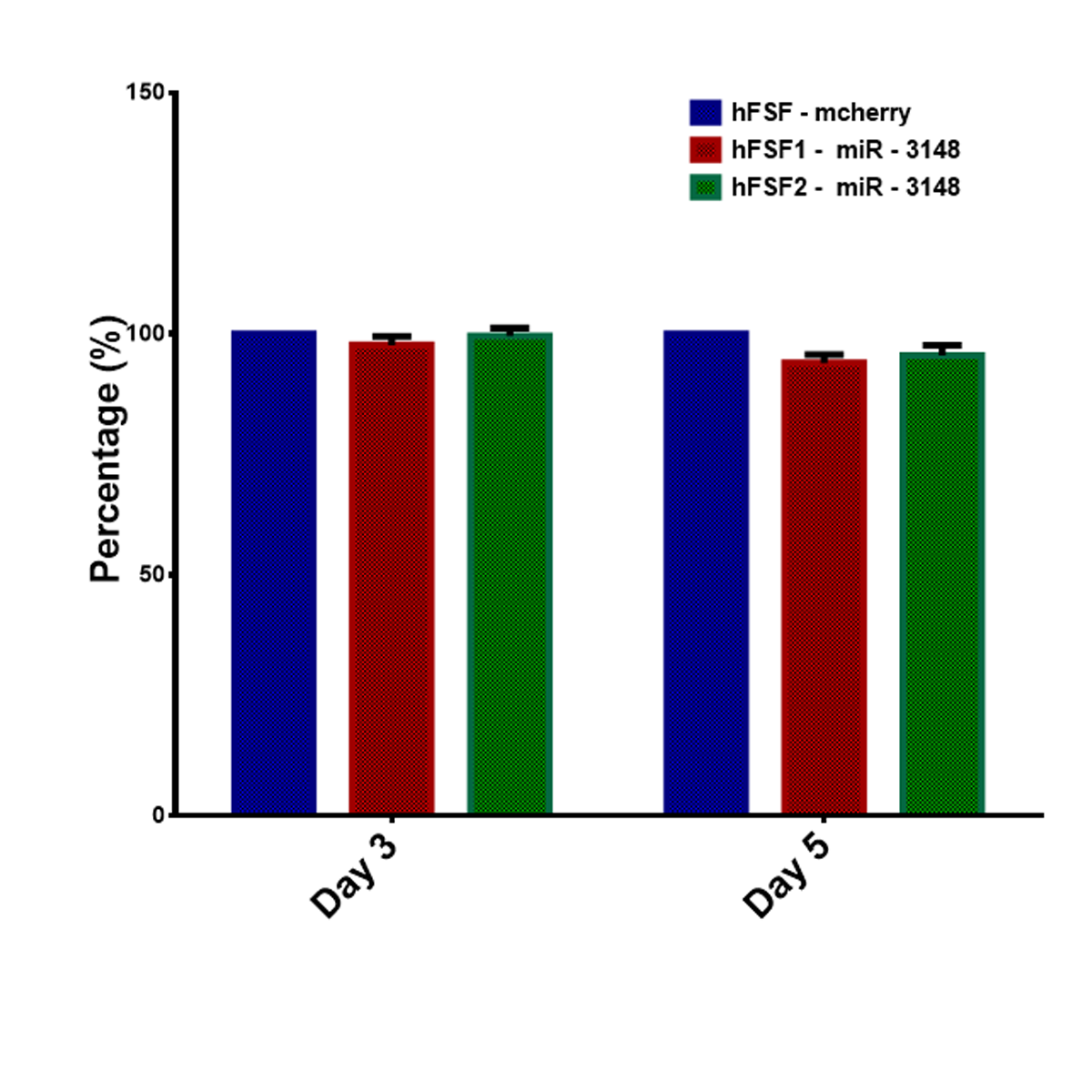

Supplement: Supplementary file 5 — Supplementary figure 5 [file 41420_2020_312_MOESM5_ESM.tif]

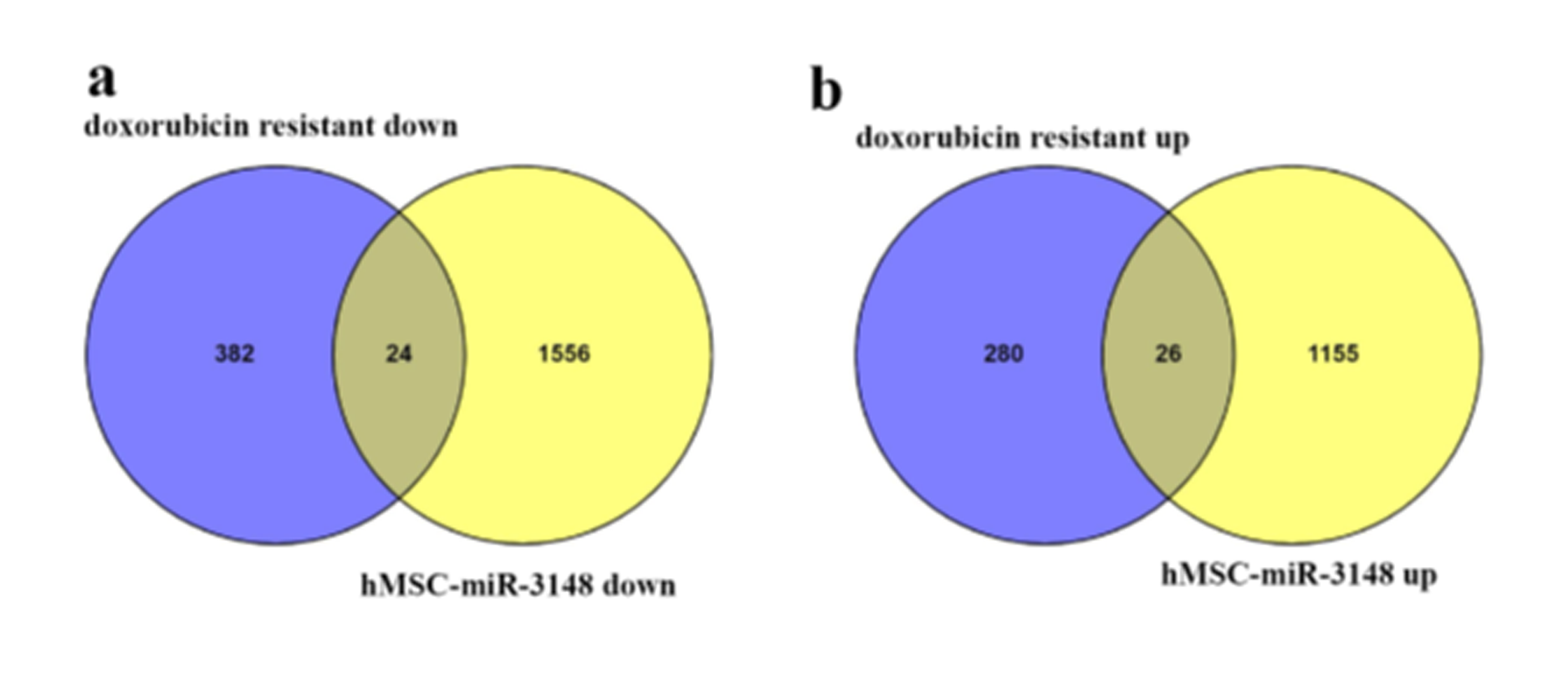

Supplement: Supplementary file 6 — Supplementary figure 6 [file 41420_2020_312_MOESM6_ESM.tif]
