# Supplementary material for: MicroRNA-3148 acts as molecular switch promoting malignant transformation and adipocytic differentiation of immortalized human bone marrow stromal cells via direct targeting of the SMAD2/TGFβ pathway
Source: Cell Death Discov. 2020 Sep 1;6:79. doi: 10.1038/s41420-020-00312-z (PMC7462980; doi:10.1038/s41420-020-00312-z)
Supplement: Supplementary file 7 — Supplementary figure and table legends [file 41420_2020_312_MOESM7_ESM.docx]

**Supplementary figure and table legends:**

**Supplementary figure 1.** MiR-3148 expression on day7 and day 13 of hMSC differentiation into osteoblast using microarray analysis.

**Supplementary figure 2.** Expression of hsa-miR-3148 in the hMSC-miR-3148 cells. qRT-PCR quantification of hsa-miR-3148 expression in hMSC-miR-3148 cells compared to hMSC-mcherry control cells. Data are representative of three experiments and are presented as mean ± S.E.M., n = 3. SNORD95 was used as endogenous control.

**Supplementary figure 3. (a)** Disease and function heat map based on IPA depicting most affected functional and disease categories according to the colour scale. Illustration of cellular movement **(b)** and cell death and survival **(c)** functional categories is presented. **(d)** IPA analysis on the downregulated gene list in hMSC-miR-3148 cells revealed remarkable inhibition of bone and connective tissue differentiation functional category. **(e)** Bar graph plot depicting the top 10 activated upstream networks based on differentially expressed genes in hMSC-miR-3148 cells based on IPA analysis.

**Supplementary figure 4.** Coomassie Brilliant Blue 2D-Gel preparative used for ID of significant spot proteins by MALDI-TOF.

**Supplementary figure 5.** Proliferation of human neonatal skin fibroblasts transduced with hsa-miR-3148 compared to cells transduced with mcherry control lentiviral vector on day3 and day5. Data are presented as mean S.D.

**Supplementary figure 6. Common upregulated and downregulated genes between hMSC-miR-3148 and doxorubicin resistance sarcoma from the GSE3362 dataset.** Gene expression data from GSE3362 was retrieved and the upregulated and downregulated genes in doxorubicin-resistant sarcoma were identified and were crossed with the list of differentially expressed genes in miR-3148-hMSC cells. Our data revealed common upregulated (ACADVL, RAG1, CYP51A1, PLAT, ITGA5, LOX, MAP4, MMP1, SCG5, STC1, THBS1, ZNF91, TNFRSF25, DDIT3, SLC16A3, SYNGR2, XRCC3, SEMA3A, POLR3G, TFPI2, KLF2, REEP2, TOR4A, STEAP3, APBB1IP, and SLC25A19) and common downregulated (CYP1B1, GJA1, IGFBP2, GSTM3, CD70, AMPH, LIF, MSX1, OAS2, THBS4, TPD52L1, BHLHE40, RUNX3, FGL1, DHRS3, FGR, SLC7A8, CD24, CELSR1, TCAF1, DACT1, PHF10, FEZ1, and C15orf48) gens.

**Supplementary table 1**. List of differentially expressed genes (2.0 FC, p <0.05) in hMSC-miR-3148 compared to hMSC-mcherry control cells.

**Supplementary table 2**. List of significant differentially expressed proteins identified in hMSC-miR-3148 compared to hMSC-mcherry cells using 2D-DIGE.
