# Supplementary material for: MicroRNA-3148 acts as molecular switch promoting malignant transformation and adipocytic differentiation of immortalized human bone marrow stromal cells via direct targeting of the SMAD2/TGFβ pathway
Source: Cell Death Discov. 2020 Sep 1;6:79. doi: 10.1038/s41420-020-00312-z (PMC7462980; doi:10.1038/s41420-020-00312-z)
Supplement: Supplementary file 9 — Supplementary Table 2 [file 41420_2020_312_MOESM9_ESM.docx]

| Supplementary table 2. List of significant differentially expressed proteins identified in hMSC-miR-3148 compared to hMSC-mcherry cells using 2D-DIGE. | | | | | | | | | | |
| --- | --- | --- | --- | --- | --- | --- | --- | --- | --- | --- |
| SpotNo^a^ | **Accession No^b^** | **Protein Name** | **MASCOT ID** | **Pi^c^** | **MW^d^** | **Cov%** | **Score** | **Ratio Treated Vs Untreated** | **P-Value**  **(ANOVA)** | **EXP^f^** |
| 1371 | [P67936](http://www.uniprot.org/uniprot/P67936) | Tropomyosin alpha-4 chain | TPM4_HUMAN | 4.67 | 28619 | 48 | 103 | 1.5 | 0.000024 | DOWN |
| 1515 | [Q9NRV9](http://www.uniprot.org/uniprot/Q9NRV9) | Heme-binding protein 1 | HEBP1_HUMAN | 5.71 | 21198 | 67 | 75 | 1.7 | 0.000053 | DOWN |
| 1609 | [P37802](http://www.uniprot.org/uniprot/P37802) | Transgelin-2 | TAGL2_HUMAN | 8.41 | 22548 | 58 | 68 | 1.5 | 0.000085 | DOWN |
| 1430 | [O75489](http://www.uniprot.org/uniprot/O75489) | NADH dehydrogenase [ubiquinone] iron-sulfur protein 3, mitochondrial | NDUS3_HUMAN | 6.99 | 30337 | 43 | 142 | 2.4 | 0.000091 | UP |
| 1662 | [Q9HCC6](http://www.uniprot.org/uniprot/Q9HCC6) | Transcription factor HES-4 | HES4_HUMAN | 11.12 | 23680 | 35 | 61 | 1.5 | 0.00028 | DOWN |
| 1244 | [P62140](http://www.uniprot.org/uniprot/P62140) | Serine/threonine-protein phosphatase PP1-beta catalytic subunit | PP1B_HUMAN | 5.84 | 37961 | 42 | 70 | 2.3 | 0.00059 | DOWN |
| 1563 | [P04179](http://www.uniprot.org/uniprot/P04179) | Superoxide dismutase [Mn], mitochondrial | SODM_HUMAN | 8.35 | 24878 | 40 | 67 | 1.7 | 0.00071 | UP |
| 1132 | [P63261](http://www.uniprot.org/uniprot/P63261) | Actin, cytoplasmic 2 | ACTG_HUMAN | 5.31 | 42108 | 56 | 84 | 1.5 | 0.001 | UP |
| 1149 | [P00558](http://www.uniprot.org/uniprot/P00558) | Phosphoglycerate kinase 1 | PGK1_HUMAN | 8.30 | 44985 | 51 | 82 | 1.7 | 0.001 | UP |
| 1439 | [Q13162](http://www.uniprot.org/uniprot/Q13162) | Peroxiredoxin-4 | PRDX4_HUMAN | 5.86 | 30749 | 61 | 130 | 1.6 | 0.002 | UP |
| 1206 | [P04075](http://www.uniprot.org/uniprot/P04075) | Fructose-bisphosphate aldolase A | ALDOA_HUMAN | 8.30 | 39851 | 53 | 93 | 1.5 | 0.002 | UP |
| 1214 | [P53004](http://www.uniprot.org/uniprot/P53004) | Biliverdin reductase A | BIEA_HUMAN | 6.06 | 33692 | 31 | 71 | 4 | 0.003 | UP |
| 576 | [P55072](http://www.uniprot.org/uniprot/P55072) | Transitional endoplasmic reticulum ATPase | TERA_HUMAN | 5.14 | 89950 | 42 | 84 | 1.5 | 0.003 | DOWN |
| 1296 | [Q15181](http://www.uniprot.org/uniprot/Q15181) | Inorganic pyrophosphatase | IPYR_HUMAN | 5.54 | 33095 | 53 | 86 | 1.5 | 0.003 | UP |
| 1396 | [Q9Y696](http://www.uniprot.org/uniprot/Q9Y696) | Chloride intracellular channel protein 4 | CLIC4_HUMAN | 5.45 | 28982 | 55 | 86 | 1.5 | 0.003 | DOWN |
| 1059 | [P07437](http://www.uniprot.org/uniprot/P07437) | Tubulin beta chain | TBB5_HUMAN | 4.78 | 50095 | 56 | 157 | 1.7 | 0.004 | DOWN |
| 1199 | [P04075](http://www.uniprot.org/uniprot/P04075) | Fructose-bisphosphate aldolase A | ALDOA_HUMAN | 8.30 | 39851 | 57 | 135 | 1.6 | 0.005 | UP |
| 1459 | [P60174](http://www.uniprot.org/uniprot/P60174) | Triosephosphate isomerase | TPIS_HUMAN | 6.45 | 26938 | 61 | 80 | 1.9 | 0.006 | UP |
| 1400 | [Q06323](http://www.uniprot.org/uniprot/Q06323) | Proteasome activator complex subunit 1 | PSME1_HUMAN | 5.78 | 28876 | 40 | 65 | 1.5 | 0.010 | UP |
| 1768 | [P05161](http://www.uniprot.org/uniprot/P05161) | Ubiquitin-like protein ISG15 | UCRP_HUMAN | 6.84 | 17933 | 48 | 75 | 3.4 | 0.01 | UP |
| 1203 | [P06132](http://www.uniprot.org/uniprot/P06132) | Uroporphyrinogen decarboxylase | DCUP_HUMAN | 5.77 | 41103 | 55 | 89 | 1.5 | 0.011 | DOWN |
| 1370 | [P07858](http://www.uniprot.org/uniprot/P07858) | Cathepsin B | CATB_HUMAN | 5.88 | 38766 | 26 | 73 | 1.5 | 0.014 | DOWN |
| 1086 | [Q9UQ80](http://www.uniprot.org/uniprot/Q9UQ80) | Proliferation-associated protein 2G4 | PA2G4_HUMAN | 6.13 | 44101 | 34 | 67 | 1.5 | 0.015 | UP |
| 1157 | [P60709](http://www.uniprot.org/uniprot/P60709) | Actin, cytoplasmic 1 | ACTB_HUMAN | 5.29 | 42052 | 62 | 88 | 1.5 | 0.015 | UP |
| 1726 | [Q9NS91](http://www.uniprot.org/uniprot/Q9NS91) | E3 ubiquitin-protein ligase RAD18 | RAD18_HUMAN | 7.17 | 57015 | 20 | 60 | 1.5 | 0.018 | DOWN |
| 1508 | [P08670](http://www.uniprot.org/uniprot/P08670) | Vimentin | VIME_HUMAN | 5.06 | 53676 | 30 | 78 | 1.5 | 0.021 | DOWN |
| 874 | [P30043](http://www.uniprot.org/uniprot/P30043) | Flavin reductase (NADPH) | BLVRB_HUMAN | 7.13 | 22219 | 72 | 98 | 1.5 | 0.021 | DOWN |
| 1891 | [P13645](http://www.uniprot.org/uniprot/P13645) | Keratin, type I cytoskeletal 10 | K1C10_HUMAN | 5.13 | 59020 | 28 | 74 | 2.3 | 0.022 | UP |
| 1092 | [P08670](http://www.uniprot.org/uniprot/P08670) | Vimentin | VIME_HUMAN | 5.06 | 53676 | 55 | 146 | 1.5 | 0.023 | DOWN |
| 1326 | [P08758](http://www.uniprot.org/uniprot/P08758) | Annexin A5 | ANXA5_HUMAN | 4.94 | 35971 | 64 | 128 | 1.5 | 0.025 | DOWN |
| 1762 | [P05161](http://www.uniprot.org/uniprot/P05161) | Ubiquitin-like protein ISG15 | UCRP_HUMAN | 6.84 | 17933 | 52 | 72 | 1.5 | 0.037 | DOWN |
| 1285 | [Q9BQE3](http://www.uniprot.org/uniprot/Q9BQE3) | Tubulin alpha-1C chain | TBA1C_HUMAN | 4.96 | 50548 | 33 | 72 | 1.5 | 0.04 | DOWN |
| 1290 | [Q15691](http://www.uniprot.org/uniprot/Q15691) | Microtubule-associated protein RP/EB family member 1 | MARE1_HUMAN | 5.02 | 30151 | 42 | 68 | 1.5 | 0.042 | DOWN |
| 1851 | [P35754](http://www.uniprot.org/uniprot/P35754) | Glutaredoxin-1 | GLRX1_HUMAN | 8.33 | 12053 | 66 | 66 | 1.6 | 0.043 | UP |
| 1272 | [P62136](http://www.uniprot.org/uniprot/P62136) | Serine/threonine-protein phosphatase PP1-alpha catalytic subunit | PP1A_HUMAN | 5.94 | 38229 | 48 | 101 | 1.5 | 0.046 | UP |
| 818 | [Q9UK05](http://www.uniprot.org/uniprot/Q9UK05) | Growth/differentiation factor 2 | GDF2_HUMAN | 6.03 | 47861 | 44 | 60 | 2.1 | 0.05 | UP |
| 913 | [P07900](http://www.uniprot.org/uniprot/P07900) | Heat shock protein HSP 90-alpha | HS90A_HUMAN | 4.94 | 85006 | 33 | 68 | 1.6 | 0.00079 | DOWN |
| 991 | [P10809](http://www.uniprot.org/uniprot/P10809) | 60 kDa heat shock protein, mitochondrial | CH60_HUMAN | 5.70 | 61187 | 53 | 107 | 1.5 | 0.002 | UP |
| 1897 | [P61088](http://www.uniprot.org/uniprot/P61088) | Ubiquitin-conjugating enzyme E2 N | UBE2N_HUMAN | 6.13 | 17184 | 74 | 94 | 1.5 | 0.003 | UP |
| 1681 | [P61081](http://www.uniprot.org/uniprot/P61081) | NEDD8-conjugating enzyme Ubc12 | UBC12_HUMAN | 7.57 | 21172 | 40 | 58 | 1.5 | 0.011 | UP |

a Spot numbers correspond to those included in the 2D-image.

b Protein accession number for SWISSPROT Database.

c Theoretical isoelectric point.

d Theoretical relative mass.

e Average ratio of differentially expressed proteins between samples.

f Protein regulation between samples.
